# Identification of an additional protein involved in mannan biosynthesis

**DOI:** 10.1111/tpj.12019

**Published:** 2012-10-19

**Authors:** Yan Wang, Jennifer C Mortimer, Jonathan Davis, Paul Dupree, Kenneth Keegstra

**Affiliations:** 1 Great Lakes Bioenergy Research Center, Michigan State UniversityEast Lansing, MI, 48824, USA; 2 Department of Energy Plant Research Laboratory, Michigan State UniversityEast Lansing, MI, 48824, USA; 3 Department of Biochemistry, University of CambridgeCambridge, CB2 1QW, UK; 4 Department of Plant Biology, Michigan State UniversityEast Lansing, MI, 48824, USA; 5 Department of Biochemistry and Molecular Biology, Michigan State UniversityEast Lansing, MI, 48824, USA

**Keywords:** mannan biosynthesis, Golgi protein, glycosyltransferase, GT65, fenugreek, Arabidopsis

## Abstract

Galactomannans comprise a β-1,4-mannan backbone substituted with α-1,6-galactosyl residues. Genes encoding the enzymes that are primarily responsible for backbone synthesis and side-chain addition of galactomannans were previously identified and characterized. To identify additional genes involved in galactomannan biosynthesis, we previously performed deep EST profiling of fenugreek (*Trigonella foenum*-*graecum* L.) seed endosperm, which accumulates large quantities of galactomannans as a reserve carbohydrate during seed development. One of the candidate genes encodes a protein that is likely to be a glycosyltransferase. Because this protein is involved in mannan biosynthesis, we named it ‘mannan synthesis-related’ (MSR). Here, we report the characterization of a fenugreek *MSR* gene (*TfMSR*) and its two Arabidopsis homologs, *AtMSR1* and *AtMSR2*. *TfMSR* was highly and specifically expressed in the endosperm. TfMSR, AtMSR1 and AtMSR2 proteins were all determined to be localized to the Golgi by fluorescence confocal microscopy. The level of mannosyl residues in stem glucomannans decreased by approximately 40% for Arabidopsis *msr1* single T-DNA insertion mutants and by more than 50% for *msr1 msr2* double mutants, but remained unchanged for *msr2* single mutants. In addition, *in vitro* mannan synthase activity from the stems of *msr1* single and *msr1 msr2* double mutants also decreased. Expression of *AtMSR1* or *AtMSR2* in the *msr1 msr2* double mutant completely or partially restored mannosyl levels. From these results, we conclude that the MSR protein is important for mannan biosynthesis, and offer some ideas about its role.

## Introduction

Mannans are hemicellulosic polysaccharides that are widespread in plants. Based on the composition of the backbone and the presence/absence of side chains, mannan polysaccharides may be categorized into four types: mannans, glucomannans, galactomannans and galactoglucomannans. Both mannans and glucomannans contain a linear β-1,4-linked backbone, with the former composed of mannosyl residues and the latter composed of both mannosyl and glucosyl residues. The mannan or glucomannan backbone may be substituted with α-1,6-linked galactosyl resisdues to produce galactomannans or galactoglucomannans (Reid, 1985). Glucomannans are the major hemicellulose in the secondary walls of gymnosperms (Fengel and Wegener, 1989). Although mannan polysaccharides are generally present at low levels in most angiosperms, they are massively deposited in certain specialized tissues of selected plants. For example, galactomannans are stored in large quantities as reserve polysaccharides specifically in the seed endosperm of many leguminous plants, including guar (*Cyamopsis tetragonoloba*) and fenugreek (*Trigonella foenum*-*graecum* L.) (Reid, 1985). Glucomannans are also stored as a reserve polysaccharide in the tubers of the voodoo lily (Gille *et al*., 2011).

Galactomannans are widely used as versatile materials in industries such as food, textiles, paper, pharmaceutics, cosmetics, petroleum, drilling and explosives (Srivastava and Kapoor, 2005). Moreover, galactomannans are entirely composed of yeast-fermentable hexoses and are easily digestible. These features make them an ideal polymer to provide sugars for biofuel production from plant biomass (Pauly and Keegstra, 2008). Understanding galactomannan biosynthesis and its regulation may ultimately make it possible to increase the level of galactomannans in plants for such practical applications.

Much has been learned about the biosynthesis of mannan polysaccharides in the last few years, in part by exploiting the systems that rapidly deposit large quantities of galactomannans (Edwards *et al*., 1999; Dhugga *et al*., 2004). Mannan polymers are synthesized in the Golgi through the action of at least two enzymes: mannan synthase (ManS) for backbone synthesis and galactomannan galactosyl transferase (GMGT) for side-chain addition. Previous studies using leguminous seeds as model systems have led to identification and characterization of mannan synthase as encoded by a *cellulose synthase-like A* (*CSLA*) gene in guar (Dhugga *et al*., 2004), and identification and characterization of the gene encoding galactomannan galactosyl transferase in fenugreek (Edwards *et al*., 1999). *CSLA* genes are found in many land plants (Yin *et al*., 2009). Heterologously expressed CSLA proteins from a variety of species show mannan or glucomannan synthase activity *in vitro* (Dhugga *et al*., 2004; Liepman *et al*., 2005; Suzuki *et al*., 2006; Liepman *et al*., 2007; Gille *et al*., 2011; Wang *et al*., 2012). Analysis of Arabidopsis *CSLA* mutants and over-expressing plants further confirmed that CSLA proteins function as glucomannan synthases *in vivo* (Goubet *et al*., 2009). Despite this progress in identifying and characterizing the enzymes responsible for galactoglucomannan biosynthesis, it is likely that other important enzymes are required, and many aspects of this process need to be better understood. For example, nothing is known about the mechanisms for initiating polymer synthesis or the ways that polymer length is determined. Polysaccharide synthesis in animals and microbes is usually initiated by formation of primer molecules in which a sugar is transferred to a protein, a lipid or even another polysaccharide (Carlsson and Kjellen, 2012; Hartley and Imperiali, 2012; Roach *et al*., 2012). The primers required for initiation of cell-wall polysaccharide synthesis in plant cells have not yet been identified.

To better understand the entire process of galactomannan biosynthesis and to begin to identify components of the transcriptional regulatory network controlling this process, deep EST profiling of fenugreek seed endosperm was performed (Wang *et al*., 2012). In this system, large quantities of galactomannans accumulate over a relatively short time frame, formed from sucrose that enters the developing seed (Wang *et al*., 2012). Using EST profiling analysis, genes likely to be involved in galactomannan biosynthesis were identified in addition to the known ManS and galactomannan galactosyl transferase genes. The second most highly expressed gene, called *TfDUF246* in the previous report (Wang *et al*., 2012) but named *TfMSR* here (for ‘mannan synthesis-related’; Genbank accession number JX237834), encodes a protein that is likely to be involved in mannan biosynthesis. Here, we have used molecular, cellular, genetic and biochemical approaches to characterize TfMSR and its two Arabidopsis homologs, AtMSR1 and AtMSR2. The results provide evidence in support of our hypothesis that these proteins are involved in mannan biosynthesis.

## Results

### *TfMSR* is specifically expressed in fenugreek seed endosperm

**Figure 1:**
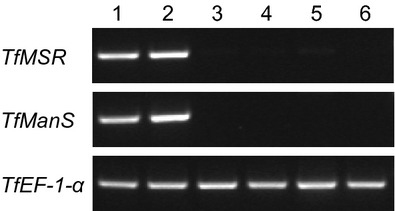
RT-PCR analysis of fenugreek *MSR* (*TfMSR*). *TfManS* was used as a reference, and fenugreek elongation factor 1α (*TfEF*-*1*-*α*) as a control. Lanes 1 and 2, fenugreek endosperms at 25 and 32 days post-anthesis; lanes 3 and 4, fenugreek embryos at 25 and 32 days post-anthesis; lanes 5 and 6, young fenugreek leaves.

The *TfMSR* full-length cDNA sequence was assembled from approximately 33 000 ESTs, and is 1454 nt long with a 1239 nt open reading frame. The deduced protein sequence is 413 amino acids long, with a predicted molecular mass of 46.4 kDa and a predicted isoelectric point of 7.89. Because *TfMSR* is highly expressed in fenugreek seed endosperm in which large quantities of galactomannans specifically accumulate, we postulated that *TfMSR* may be involved in galactomannan biosynthesis. If so, then *TfMSR* expression should be limited to the endosperm. RT-PCR analysis using RNA isolated from various fenugreek tissues did indeed show specific expression of *TfMSR* in the endosperm, as is the case for the fenugreek *ManS* (*TfManS*) gene (Genbank accession number JX237835) (Figure 1). Both genes are among the ten most highly expressed genes in the endosperm (Wang *et al*., 2012).

### Identification of Arabidopsis homologs

Because a method for fenugreek transformation has not been established, and genetic tools are not available, it is very difficult to perform molecular genetic analysis of *MSR* function in fenugreek. Therefore, we sought to identify Arabidopsis homologs so that the power of Arabidopsis molecular genetics could be used in functional characterization of this gene. The deduced amino acid sequence of TfMSR was used as a query to search against the Arabidopsis protein database (http://www.arabidopsis.org/) using BLASTP. This analysis yielded two homologs, At3g21190 and At1g51630, which were called AtMSR1 and AtMSR2, respectively. TfMSR shows 47 and 45% sequence identity, and 67 and 65% sequence similarity, to AtMSR1 and AtMSR2, respectively, and AtMSR1 shows 83% sequence identity and 91% sequence similarity to AtMSR2 (Figure S1). The three proteins also have similar sizes (413, 422 and 423 amino acids) and were predicted to have a transmembrane domain at the N-terminus and a large conserved domain at the C-terminus (Figure S1). Because AtMSR1 and AtMSR2 are homologs of TfMSR, we postulated that they may also be involved in mannan biosynthesis.

### TfMSR, AtMSR1 and AtMSR2 are localized to the Golgi body

**Figure 2:**
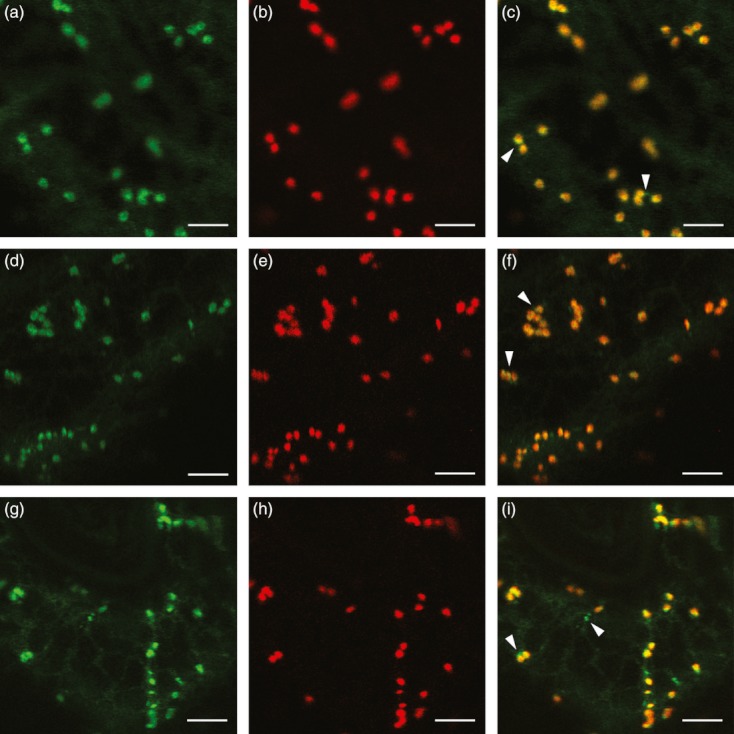
Cellular localization of GFP-tagged MSR (GFP–MSR) proteins. (a) GFP–TfMSR; (d) GFP–AtMSR1; (g) GFP–AtMSR2; (b, e, h) ST-mRFP; (c, f, i) merged images. Arrowheads indicate punctate structures where the ST-mRFP signal was not present or did not completely overlap with the GFP–MSR signal. Scale bar = 5 μm.

If MSR proteins are directly involved in mannan biosynthesis, they should be localized to the Golgi apparatus where mannans and other matrix polysaccharides are synthesized. Using proteomic techniques, Dunkley *et al*. (2004, 2006) and Parsons *et al*. (2012) previously reported that AtMSR1 and AtMSR2 were localized to the Golgi apparatus. To examine the localization of TfMSR and confirm the localization of its two Arabidopsis homologs, a GFP-tagged version of each protein was transiently expressed together with a Golgi marker (ST-mRFP) (Renna *et al*., 2005) in tobacco leaves, and the localization was examined using live-cell confocal microscopy. As expected, GFP-tagged MSR proteins co-localized with the Golgi marker ST-mRFP in punctate structures (Figure 2), indicating their localization in the Golgi. GFP-tagged MSR proteins were also found in some punctate structures where the ST-mRFP signal was not present or did not completely overlap with the GFP signal. Similar patterns showing incomplete overlap of signal were also found in a localization study for AtCSLA9 and AtCSLC4 (Davis *et al*., 2010), and may indicate localization to the *trans*-Golgi network or secretory structures outside the Golgi proper.

### Expression of *AtMSR1* and *AtMSR2*

**Figure 3:**
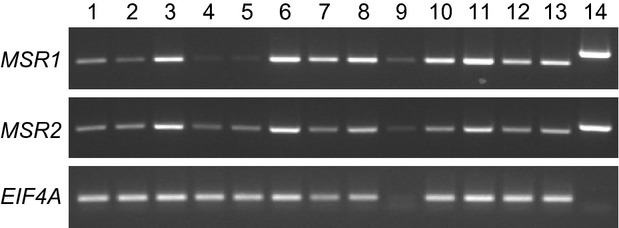
RT-PCR analysis of Arabidopsis *MSR* genes (*AtMSR1* and *AtMSR2*). Lane 1, whole seedlings; lane 2, hypocotyls + cotyledons of seedlings; lane 3, seedling roots; lane 4, rosette leaves; lane 5, cauline leaves; lane 6, flowers; lane 7, siliques without seeds; lane 8, siliques with seeds; lane 9, seeds; lane 10, whole stem; lanes 11–13, top, mid and basal thirds of a stem; lane 14, genomic DNA as a control. All RNA and genomic DNA were isolated from wild-type Col-0 plants (8 days old for lanes 1–3 and lane 14, and 7 weeks old for lanes 4–13). *EIF4A*, the Arabidopsis translation initiation factor 4A-1 gene (*AtEIF4A1*, *At3g13920*), was used as a control.

We first examined expression of *AtMSR1* and *AtMSR2* in various tissues of wild-type (WT) Col-0 plants by RT-PCR (Figure 3). Both genes were expressed in all tissues examined, and appeared to have higher transcript levels in seedling roots, flowers, siliques and stems, particularly the top region of the stem (Figure 3).

**Figure 4:**
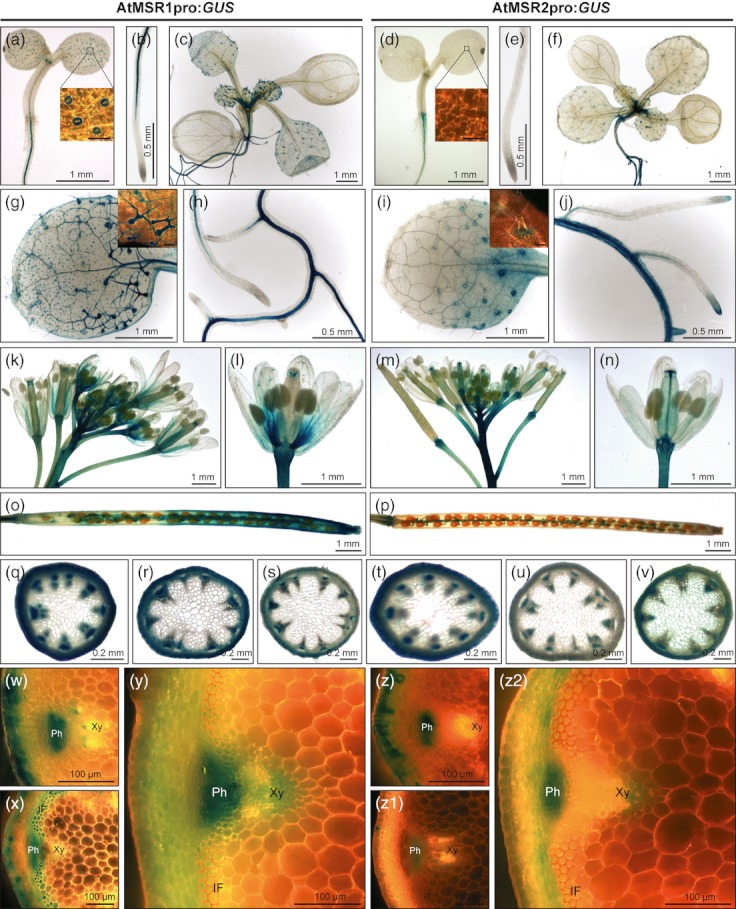
Tissue-specific expression of AtMSR_pro_:GUS. (a, d) Four-day-old seedling. (b, e) Root of a 4-day-old seedling. (c, f) Ten-day-old seedling. (g, i) Young leaf of a 10-day-old seedling. (h, j) Lateral roots of a 10-day-old seedling. (k, m) Flower bundle of a 7-week-old plant. (l, n) Young flower. (o, p) Silique of a 7-week-old plant. (q–v) Transverse sections at top (q, t), mid (r, u) and basal (s, v) regions of the primary stem of a 7-week-old AtMSR_pro_:GUS plant (photographs taken under a stereoscope). (w, x, y) Photographs of the same sections as (q), (r) and (s) taken under a light microscope with a dark-field filter. (z, z1, z2) Photographs of the same sections as (t), (u) and (v) taken under a light microscope with a dark-field filter. IF, inter-fascicular fiber; Ph, phloem; Xy, xylem. Scale bars = 50 μm for inserts to (a) and (d) showing cotyledon epidermis and 100 μm for inserts to (g) and (i) showing leaf trichomes.

To further study tissue-specific expression of the two genes, stable transgenic lines containing AtMSR_pro_:GUS transcriptional fusion constructs were generated. GUS staining of whole seedlings or various tissues of the transgenic lines revealed overlapping and also different or complementary expression patterns of *AtMSR1* and *AtMSR2* (Figure 4). In young seedlings, both genes were highly expressed in leaf primordia, young leaves and roots. For *AtMSR1*, strong GUS staining was found in the vascular tissue of roots except the basal region, and for *AtMSR2*, strong GUS staining was found in the vascular tissue and epidermis of roots as well as tips of young lateral roots, but not in the basal half of the primary root of 4-day-old seedlings or the region between the upper half and the tip of lateral roots. *AtMSR1* was also highly expressed in guard cells of cotyledons as well as trichomes, veins (vascular tissue) and guard cells of young leaves, whereas *AtMSR2* was highly expressed in a ring of cells (called socket cells or accessory cells) surrounding the base of trichomes. The expression of both genes was higher in the proximal half of young leaves than in the distal half.

Both genes were also highly expressed in flowers, siliques and stems of mature plants, with higher levels in younger organs. Strong GUS staining was found in petioles and petals of flowers for *AtMSR1*, and in petioles and pistils of flowers for *AtMSR2*. In stems, both genes were expressed strongly in the phloem and weakly in the xylem and epidermis. In the middle region of stems, strong *AtMSR1* expression was also found in inter-fascicular fiber cells. The two genes showed higher expression in the top region of stems than in the basal region.

The expression data for *AtMSR1* and *AtMSR2* based on GUS staining are consistent with the data from our RT-PCR analysis. In addition, the strong expression of *AtMSR1* in stems, siliques and flowers as well as trichomes and guard cells was consistent with previously published microarray data (Figure S2). No microarray data are available for *AtMSR2*.

### Mutations in *AtMSR* lead to reduction of mannan levels

**Figure 5:**
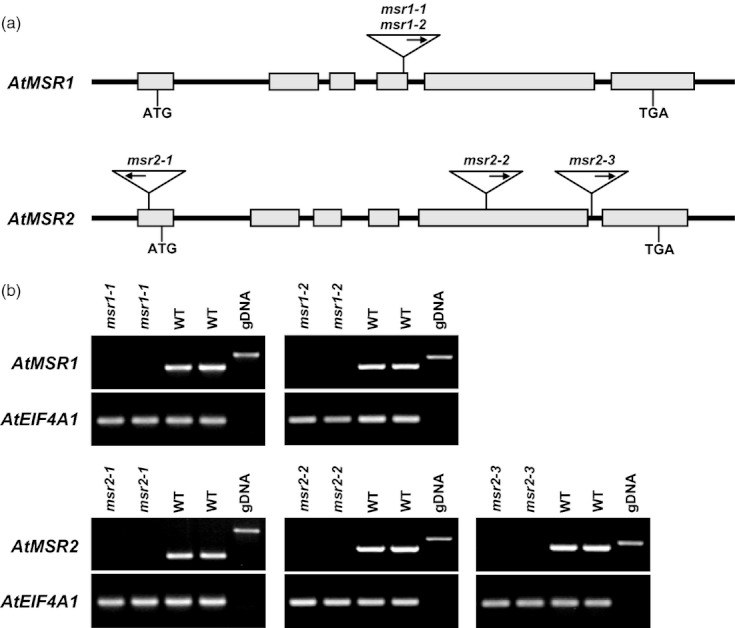
Identification of T-DNA insertion lines for two *AtMSR* genes. (a) Gene structures and T-DNA insertion sites. Arrows indicate the orientation of T-DNA insertions from the right border to the left border. (b) RT-PCR analysis of the T-DNA insertion lines using RNA isolated from primary stems. gDNA, genomic DNA isolated from wild-type Col-0 plants. *AtEIF4A1* (*At3g13920*) was used as a control.

To investigate the function of the two *AtMSR* genes, T-DNA insertion lines were obtained: SALK_138965 (*msr1*-*1*) and SALK_075245 (*msr1*-*2*) for *AtMSR1*, and GK-017H03 (*msr2*-*1*), SAIL_576_E11 (*msr2*-*2*) and SAIL_46_F12 (*msr2*-*3*) for *AtMSR2*. For *msr1*-*2*, the T-DNA was annotated as inserted in the fourth intron (http://www.arabidopsis.org/), but our sequencing analysis revealed that the T-DNA insertion was actually located in the fourth exon, very close to that for *msr1*-*1* (Figure 5a). All lines were confirmed to be true transcriptional knockouts by RT-PCR (Figure 5b). Two double mutants (*msr1*-*1 msr2*-*1* and *msr1*-*1 msr2*-*2*) were generated by crossing *msr1*-*1* with *msr2*-*1* or *msr2*-*2*. All single and double mutant plants looked morphologically normal. Because *AtMSR1* was highly expressed in trichomes and guard cells and *AtMSR2* was highly expressed in socket cells surrounding the base of trichomes, we also carefully examined leaf trichomes and guard cells of the mutants under a scanning electronic microscope, but no obvious phenotype was found.

**Figure 6:**
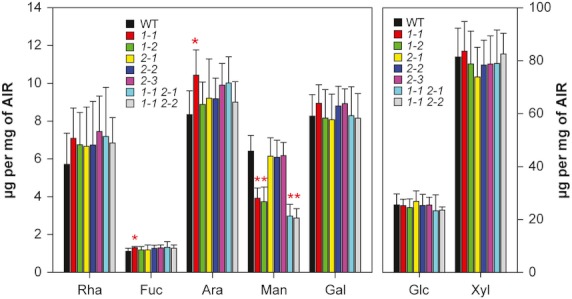
Non-cellulose neutral monosaccharide composition of primary stems from 6-week-old plants. Error bars represent the standard deviation of three biological replicates, with three technical replicates for each. Asterisks indicate statistically significant differences relative to the wild-type (Student's *t* test, *P* < 0.01). The *P* values for the mannosyl level of *msr1*-*1*, *msr1*-*2*, *msr1*-*1 msr2*-*1* and *msr1*-*1 msr2*-*2* are less than 3 × 10^−6^. *1*-*1*, *msr1*-*1*; *1*-*2*, *msr1*-*2*; *2*-*1*, *msr2*-*1*; *2*-*2*, *msr2*-*2*; *2*-*3*, *msr2*-*3*; *1*-*1 2*-*1*, *msr1*-*1 msr2*-*1* double mutant; *1*-*1 2*-*2*, *msr1*-*1 msr2*-*2* double mutant; WT, Col-0 wild-type. Ara, arabinose; Fuc, fucose; Gal, galactose; Glc, glucose; Man, mannose; Rha, rhamnose; Xyl, xylose.

To determine whether *AtMSR* genes are involved in mannan biosynthesis, neutral monosaccharide composition analysis was performed using alcohol-insoluble residue (AIR) from the primary stems of WT and *msr* mutant plants (Figure 6). The mannosyl levels decreased by approximately 40% in *msr1*-*1* and *msr1*-*2* single mutants, and further decreased to less than 50% of WT levels in the double mutants. However, no significant changes in the mannosyl levels were found in the three *msr2* single mutants. Despite differences in fucosyl and arabinosyl levels found between *msr1*-*1* and the WT, the results were not consistent in that the differences were not found in *msr1*-*2* or the double mutants. In addition, the level of fucosyl residues was very low, and this low signal may contribute to the poor signal/noise ratio. We also analyzed the crystalline cellulose content in the stems of the mutants, and found no significant changes.

**Figure 7:**
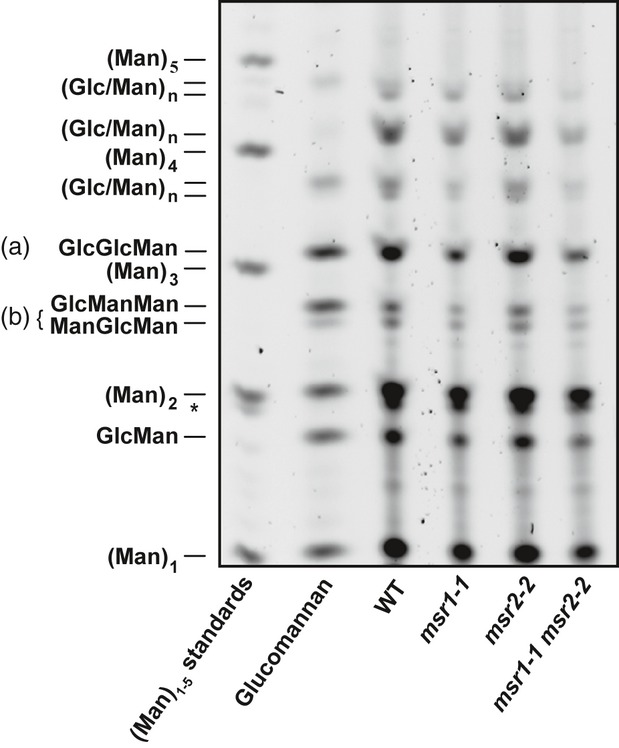
Polysaccharide analysis by carbohydrate gel electrophoresis of mannan polysaccharides in the cell wall of Arabidopsis primary stems. Cell walls [(alcohol-insoluble residue (AIR)] were digested using mannanases, and the resulting oligosaccharides were derivatized using 2-aminonaphthalene trisulfone followed by separation by polyacrylamide gel electrophoresis. Konjac glucomannan treated in the same way is shown for comparison. The asterisk indicates a non-specific band from a compound in the reaction reagents, and the bands quantified in Figure S3 are labeled ‘a’ and ‘b’. A representative gel is shown; the experiment was performed on three biological replicates, with two technical replicates of each. Man, mannose; Glc, Glucose.

We next used PACE (polysaccharide analysis by carbohydrate gel electrophoresis) to examine whether the reduction in the level of mannosyl residues was due to a decrease in mannan content. AIR from the primary stems of WT, *msr1*-*1*, *msr2*-*2* and *msr1*-*1 msr2*-*2* was digested using mannanases. The reducing ends of the released oligosaccharides were derivatized with a fluorophore, and separated by gel electrophoresis (Figure 7). This technique may be used to identify the composition and structure of the mannan polysaccharide, because oligosaccharides show characteristic migration within the gel (Goubet *et al*., 2002; Handford *et al*., 2003). In addition, because each fluorescent molecule represents a single reducing end, relative quantification may be performed within the gel to compare samples (Goubet *et al*., 2009). Consistent with the monosaccharide composition analysis, although *msr2*-*2* showed no significant difference in glucomannan content compared to WT by PACE, *msr1*-*1* showed an approximately 50% reduction (Figures 7 and S3). The double mutant had less glucomannan than *msr1*-*1* alone, suggesting some redundancy of function for these two genes.

**Figure 8:**
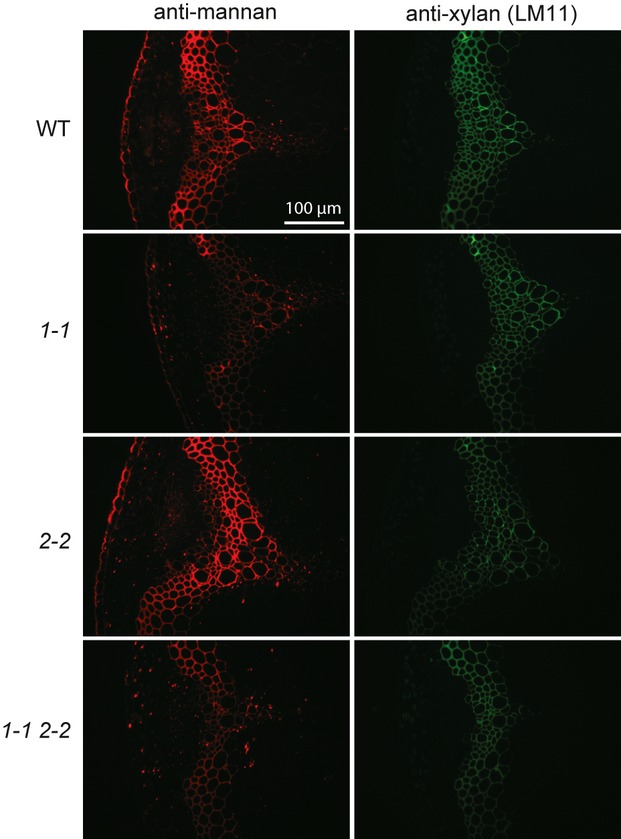
Immunofluorescence labeling of Arabidopsis basal stem sections. Sections were labeled with antibodies specific for mannan (Handford *et al*., 2003) and xylan (LM11; McCartney *et al*., 2005). Scale bar = 100 μm. Images for individual antibodies were taken with the same exposure for all sections. Three technical replicates were performed on sections from two biological replicates. Representative images are shown. *1*-*1*, *msr1*-*1*; *2*-*2*, *msr2*-*2*; *1*-*1 2*-*2*, *msr1*-*1 msr2*-*2* double mutant.

In order to test whether the reduction in glucomannan shows cell specificity within the stem, immunofluorescent labeling using an antibody specific to mannan was performed on sections from mature Arabidopsis stems (Figure 8). WT stems showed strong mannan labeling in the walls of xylem vessels and epidermal cells, as previously reported using this antibody (Handford *et al*., 2003; Goubet *et al*., 2009). *msr1*-*1* showed a general decrease in anti-mannan labeling, whereas *msr2*-*2* labeling resembled that of the WT. In multiple experiments, *msr1*-*1 msr2*-*2* clearly showed less labeling than *msr1*-*1*, particularly in the external walls of the epidermal cells. However, it should be noted that the *AtMSR* expression patterns do not always match the mannan deposition detected; for example, both genes are very highly expressed in the phloem tissue (Figure 4), but very little mannan is detected in these cells in the WT (Figure 8). Immunolabeling of xylan was also performed in order to confirm that the reduction in labeling was mannan-specific (Figure 8).

### Microsomes from *msr1-1* and double mutants showed lower mannan synthase activity *in vitro*

To explore the reason for mannan reduction in the *msr1* single mutants and the *msr1*-*1 msr2*-*1* and *msr1*-*1 msr2*-*2* double mutants, quantitative RT-PCR was performed. We first checked the expression level of *AtMSR1* and *AtMSR2* in *msr1* and *msr2* single mutants. Although transcripts of *AtMSR1* or *AtMSR2* in the corresponding single mutants were not detected using primers flanking the T-DNA insertion sites (Figure 5b), the expression level of *AtMSR1* in the *msr2* mutants and of *AtMSR2* in the *msr1*-*2* mutant was similar to that in the WT, and *AtMSR2* showed slightly higher expression in the *msr1*-*1* mutant than in the WT (Figure S4a).

We then examined the expression levels of four *AtCSLA* genes in the *msr* double mutants. *AtCSLA2*, *AtCSLA3* and *AtCSLA9* have been shown to be required for glucomannan synthesis in the stem of Arabidopsis (Goubet *et al*., 2009). *AtCSLA10* was also studied because the expression level of *AtCSLA10* in the stem was twice as high as that of *AtCSLA3*, but lower than that of *AtCSLA2* and *AtCSLA9* (Liepman *et al*., 2007). All four genes were expressed at a higher level in the double mutants than in the WT, with expression levels of *AtCSLA2* and *AtCSLA9* being significantly higher than that in the WT (Figure S4b). One interpretation of these results is that there may be a compensation mechanism in the double mutants, and that mannan reduction in the *msr* mutants was not due to change in the transcript levels of *AtCSLA* genes.

**Figure 9:**
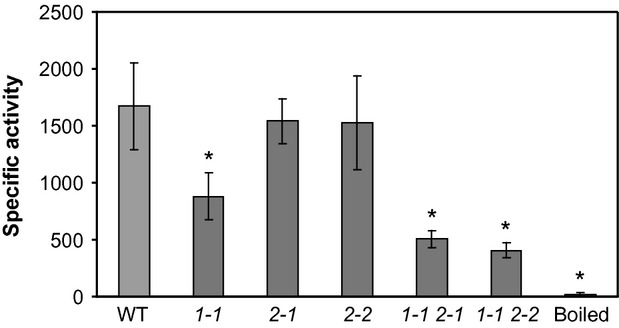
*In vitro* mannan synthase activity assay of microsomes prepared from stems. Error bars represent the standard deviation of three biological replicates, with three technical replicates for each. The specific activity is shown as pmol GDP-Man incorporation per hour per mg protein. Boiled, boiled wild-type control. Asterisks indicate statistically significant differences relative to the wild-type (Student's *t* test, *P* < 3 × 10^−8^). *1*-*1*, *msr1*-*1*; *2*-*1*, *msr2*-*1*; *2*-*2*, *msr2*-*2*; *1*-*1 2*-*1*, *msr1*-*1 msr2*-*1* double mutant; *1*-*1 2*-*2*, *msr1*-*1 msr2*-*2* double mutant.

To test whether mannan reduction in the *msr* mutants occurred at the enzymatic activity level, ManS activity assays using GDP-[^14^C]-Man and endogenous acceptors were performed using microsomes prepared from the whole stem of each WT or mutant plant. As shown in Figure 9, the *in vitro* ManS activity decreased by 47% for *msr1*-*1*, and by 70% or more for the *msr1*-*1 msr2*-*1* and *msr1*-*1 msr2*-*2* double mutants, compared with the WT. However, no significant change was found for the *msr2*-*1* and *msr2*-*2* single mutants. Therefore, the changes in the *in vitro* ManS activity are consistent with alterations in the levels of mannosyl residues and glucomannan.

### Expression of *AtMSR1* or *AtMSR2* completely or partially restored the mannosyl level in the *msr1-1 msr2-2* double mutant

**Figure 10:**
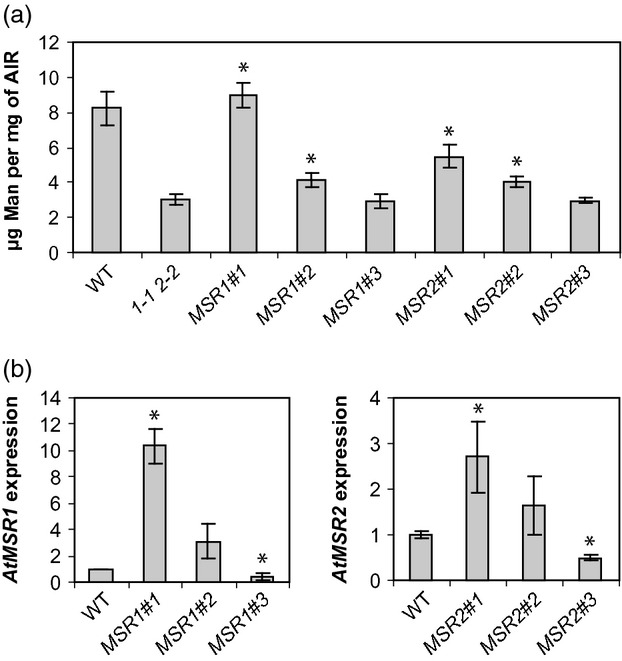
Expression of *AtMSR1* or *AtMSR2* rescued the mannosyl reduction phenotype of the *msr1*-*1 msr2*-*2* double mutant. (a) Mannosyl levels in the primary stems of *AtMSR-*expressing lines (in the *msr1*-*1 msr2*-*2* background) in comparison with the WT and the *msr1*-*1 msr2*-*2* (*1*-*1 2*-*2*) double mutant. Error bars represent the standard deviation of four biological replicates, with three technical replicates for each. Asterisks indicate statistically significant differences relative to the *msr1*-*1 msr2*-*2* double mutant (Student's *t* test, *P* < 2 × 10^−8^).(b) Expression level of the *AtMSR* transgene in the primary stems of expressing lines. The expression level of *AtMSR* was normalized to that of the reference gene *At4G26410*, and is presented relative to the WT level. Error bars represent the standard deviation of three biological replicates, with two technical replicates for each. Asterisks indicate statistically significant differences relative to the WT (Student's *t* test, *P* < 0.02). *MSR1#1*, *MSR1#2* and *MSR1#3*, three *MSR1*-expressing lines; *MSR2#1*, *MSR2#2* and *MSR2#3*, three *MSR2-*expressing lines.

To confirm that the reduction in mannan levels in the mutant plants was caused by the mutations in the *MSR* genes, complementation experiments were performed. *AtMSR1* or *AtMSR2* was used to complement the *msr1*-*1 msr2*-*2* double mutant, and the primary stems of the complemented lines were subject to neutral sugar analysis. Expression of the genes under the control of the 35S promoter partially or completely rescued the reduced mannosyl levels observed in the double mutant. The mannosyl level was slightly higher than that of the WT for one of the *AtMSR1*-expressing lines, and between that of the WT and the double mutant for another *AtMSR1*-expressing line and two *AtMSR2*-expressing lines (Figure 10a). The mannosyl levels of the complemented lines correlated with the transcript levels of the transgenes in these lines (Figure 10). There was no significant difference in the levels of other neutral sugars between the complemented lines and the WT, or between these lines and the double mutant.

We attempted to complement the Arabidopsis *msr1*-*1 msr2*-*2* double mutant using the fenugreek *MSR* gene under the control of the 35S promoter. Ten independent transgenic lines were characterized, but the mannosyl reduction phenotype was not rescued in any of the lines. The expression levels of the *TfMSR* transgene in these lines were very low (at least 20 times lower than that of *AtMSR1* or *AtMSR2* in the WT), and this may explain the inability of *TfMSR* to complement the double mutant.

### Genes related to *MSR* are present as a large family in all land plants

Shortly after the discovery of the *TfMSR* gene (Wang *et al*., 2012), a search of the Pfam database (Finn *et al*., 2010) revealed only one predicted domain, DUF246 (Pfam identity number PF03138), which is present in the C-terminal half of TfMSR. DUF246 was defined as a plant-specific protein domain (Hansen *et al*., 2012). The DUF246 domain was later merged into the GDP-fucose protein *O*-fucosyltransferase (O-FucT) domain (Pfam identity number PF10250) (Hansen *et al*., 2012) because several Arabidopsis DUF246 proteins were predicted by Hansen *et al*. (2009) to be glycosyltransferase (GT) candidates distantly related to family GT65 in the CAZy database (Coutinho *et al*., 2003). In animals, O-FucT proteins add a fucose residue directly to serine or threonine residues of proteins (Ma *et al*., 2006). A structural homology search using the FUGUE program (Shi *et al*., 2001) revealed that TfMSR showed structural similarity to a *Caenorhabditis elegans* O-FucT (CePOFUT1; PDB ID 3zy2; Lira-Navarrete *et al*., 2011) (ZSCORE = 10.23; a ZSCORE ≥ 6 means 99% prediction confidence). Although a clear GT signature related to GT65 was found in plant DUF246 or putative O-FucT proteins, several amino acids that are important for O-FucT activity in animals are not strictly conserved in these plant proteins, suggesting that these plant proteins may use a substrate other than GDP-fucose (Hansen *et al*., 2012). Because plant putative O-FucTs were annotated based on animal data (Parsons *et al*., 2012) and none has been proven to have O-FucT activity, we propose that MSR proteins and other annotated plant putative O-FucTs should be called GT65R proteins.

By searching available sequenced genomes using the TfMSR protein sequence as a query or the domain identity number ‘PF10250’ as a key word, *GT65R* genes were found in all land plants including moss, Arabidopsis and rice, but were absent in the genomes of five unicellular and one multicellular green algae (Table S1). In Arabidopsis, 39 genes currently annotated as putative O-FucT genes were identified; 34 of them were previously annotated as DUF246 genes (Table S2).

All 39 Arabidopsis sequences annotated as putative O-FucTs contain a conserved domain at the C-terminal half, and almost all of them were predicted to contain one transmembrane domain at or near the N-terminus. All Arabidopsis GT65R proteins were predicted to show structural similarity to CePOFUT1 (Lira-Navarrete *et al*., 2011), like TfMSR. The cellular localizations for 14 of them have been experimentally determined, mainly by proteomic approaches, and ten were located in the Golgi (Table S2).

Due to great variations in the N-terminal region, only sequences of the conserved domain of the 39 Arabidopsis GT65R proteins were used for phylogenetic analysis. These GT65R proteins show high sequence diversity and cluster into different clades (Figure S5).

## Discussion

Here we present evidence in support of the conclusion that *MSR*, identified from a previous EST profiling analysis of fenugreek endosperm, is involved in mannan biosynthesis. This conclusion is consistent with evidence from MSR gene expression and protein localization data from both fenugreek and Arabidopsis. First, *TfMSR* is specifically expressed in the endosperm of fenugreek seeds (Figure 1), where high levels of galactomannan deposition occur (Reid, 1985; Wang *et al*., 2012). Second, TfMSR and its two homologous Arabidopsis proteins (AtMSR1 and AtMSR2) are localized to the Golgi (Figure 2) (Dunkley *et al*., 2004, 2006; Parsons *et al*., 2012) where glucomannans are synthesized. Third, *AtMSR1* and *AtMSR2* are highly expressed in flowers, siliques and stems (Figures 4 and S2a), consistent with higher mannan content in these tissues (Moller *et al*., 2007). Fourth, the high-level expression of *AtMSR1* in guard cells and trichomes (Figures 4 and S2) is consistent with that of *AtCSLA2*, *AtCSLA3* and *AtCSLA9* in guard cells and of *AtCSLA9* in trichomes (Figure S2). The cell-specific expression pattern of *AtMSR1* is consistent with the high mannosyl level (22 μg mg^−1^ AIR or 15% of the total matrix neutral sugars) in trichomes (Marks *et al.*, 2008) compared with our sugar composition data (6.4 μg mg^−1^ AIR or 4.7% of the total matrix neutral sugars) from Arabidopsis stems (Figure 6).

More direct evidence for a role for MSR protein in mannan biosynthesis comes from analysis of Arabidopsis *AtMSR* knockout mutants. Composition analysis revealed that the mannosyl levels of AIR (mainly cell walls) decreased by approximately 40% in the stems of *msr1* single mutants, and by more than 50% in the stems of *msr1*-*1 msr2*-*1* and *msr1*-*1 msr2*-*2* double mutants (Figure 6). PACE and immunolabeling analyses demonstrated that the reduction in the mannosyl level was the result of a decreased level of cell-wall glucomannans in the stems of mutant plants (Figures 7 and 8). *In vitro* activity assays using isolated microsomes derived from the stems of mutant plants showed a significant reduction in ManS activity (Figure 9). Finally, the reduction in mannosyl levels in the *msr1*-*1 msr2*-*2* double mutant plants was completely or partially rescued by expressing *AtMSR1* or *AtMSR2* under the control of the 35S promoter (Figure 10). In the complemented lines, the mannosyl levels correlated with the transcript levels of the transgenes (Figure 10). The observation that higher transcript levels of each gene were required to achieve full complementation probably reflects the fact that the constitutive 35S promoter drove expression of the *MSR* genes in many other cell types than those where they are normally expressed.

AtMSR1 was generally more important for mannan biosynthesis than AtMSR2. The reductions in the levels of mannosyl residues, mannans and ManS activity in stems of *msr2* single mutant plants were much less than those in stems of *msr1* single mutant plants (Figures 9). However, further reductions in these levels occurred in the double mutants compared with the *msr1* single mutants, leading us to conclude that AtMSR2 also plays a role in mannan biosynthesis. Based on these results, we conclude that, although AtMSR1 and AtMSR2 may function similarly in mannan biosynthesis, AtMSR1 plays a major role whereas AtMSR2 may play a subsidiary role in maintaining full mannan biosynthetic activity in Arabidopsis. The difference in the levels of mannans and ManS activity between the *msr1* and *msr2* single mutants may also be due to different cell-specific expression patterns of the genes (Figure 4).

The reduction in ManS activity in the *msr1*-*1* single mutant and the double mutants provides evidence for a role of the MSR proteins in mannan biosynthesis (Figure 9). CSLA proteins have been shown to synthesize mannans or glucomannans *in vitro* when heterologously expressed in insect and *Pichia* cells (Liepman *et al*., 2005, 2007). Because MSR proteins are not present in these heterologous systems, we conclude that they are not required for mannan formation, at least *in vitro*. Because it is known that CSLA proteins are responsible for mannan synthase activity (Dhugga *et al*., 2004; Liepman *et al*., 2005, 2007; Goubet *et al*., 2009), it is noteworthy that the expression of *CSLA* genes increased in both *msr* double mutants (Figure S4b). This observation led us to speculate that reduced levels of mannan synthase activity result in increased *CSLA* expression in an effort to increase mannan synthase activity.

An important unresolved issue is the nature of the biochemical function of the MSR proteins in mannan biosynthesis. Despite the evidence provided in this work that MSR proteins are involved in mannan biosynthesis, the fact that they lack significant sequence similarity to proteins of known function makes it difficult to predict their precise biochemical role. However, based on clues from bioinformatic predictions and related arguments, several hypotheses may be proposed. First is the possibility that MSR proteins are GTs that are involved in producing the primers needed to initiate mannan biosynthesis. One compelling piece of evidence that MSR and related proteins in the GT65R family are GTs is their structural similarity to CePOFUT1 (Lira-Navarrete *et al*., 2011). In this scenario, one important unresolved issue is whether the acceptor molecular for the GT activity is a protein, a lipid or another carbohydrate. All are known to function as primers for polysaccharide biosynthesis in other systems (Carlsson and Kjellen, 2012; Hartley and Imperiali, 2012; Roach *et al*., 2012). This hypothesis is consistent with the localization and predicted topology of the MSR proteins. All of the MSR proteins were located in the Golgi (Figure 2) (Dunkley *et al*., 2004, 2006; Parsons *et al*., 2012) where most glycosylation reactions occur (Breton *et al*., 2006), and were predicted to have the same topology (Figure S1) as Golgi-localized GTs, which have a large C-terminal globular catalytic domain facing the lumen (Breton *et al*., 2006) and typically have a single transmembrane helix (Hansen *et al*., 2012). If this hypothesis is correct, then other primers may function in Arabidopsis when both *MSR* genes are disrupted; it is possible that other members of the GT65R family of proteins function to prime the synthesis of other polysaccharides and may function at lower efficiency to prime mannan synthesis when the MSR proteins are not present. It is also possible that other molecules function as primers when *CSLA* genes are expressed in heterologous systems, thereby allowing *in vitro* synthesis of mannans in these systems.

A second possibility is that MSR proteins may function in the synthesis of oligosaccharides linked to ManS or ManS-interacting proteins to promote folding, stability or activity of the complex. The second scenario is consistent with the presence of at least one predicted *N*-glycosylation site in CSLA proteins as well as the localization data and predicted topology described in the first hypothesis. Third, the MSR protein may not function as a GT, but rather may interact directly or indirectly with ManS to confer stability or enhance the activity of ManS. This possible interaction is supported by the same localization (in the Golgi lumen) of the active site-containing large globular region of AtCSLA9 (Davis *et al*., 2010), and the predicted large conserved C-terminal domain of MSR proteins (Figure S1). All three hypotheses are consistent with reduced ManS activity in the *msr* mutants (Figure 9).

The two Arabidopsis *MSR* genes are related to a family of genes referred to as *GT65R* genes. This family of genes is only found in land plants, including moss, Arabidopsis and rice. *GT65R* genes are absent from five unicellular green algae and one multicellular green algae (*Volvox carteri*) (Table S1), which contain only a single-copy *CSL* gene (Yin *et al*., 2009) that is closely related to both *CSLA* and *CSLC* (*cellulose synthase-like C* ); the latter encodes xyloglucan glucan synthase (Cocuron *et al*., 2007). These green algae either lack cell walls or have cell walls consisting of hydroxyproline-rich proteins (Miller *et al*., 1974; Chretiennotdinet *et al*., 1995; Worden *et al*., 2009), whereas land plants, including mosses, have cell walls containing many types of polysaccharides, including mannans of various types (Popper and Fry, 2003; Liepman *et al*., 2007; Popper *et al*., 2011). Therefore, the presence of *GT65R* genes correlates with the existence of complex walls containing many types of polysaccharides.

Arabidopsis has 39 genes that are annotated as being in the GT65R family. These Arabidopsis GT65R proteins show high sequence diversity and cluster into different clades (Hansen *et al*., 2012), suggesting that they may have evolved related but different functions (Hansen *et al*., 2012). Ten of the GT65R proteins (including AtMSR1 and AtMSR2) have been experimentally demonstrated to be Golgi-localized proteins (Figure 2 and Table S2). Because almost all GT65R proteins were predicted to have the same topology (Figure S1 and Table S2), we speculate that most (if not all) of these proteins are localized to the Golgi where most GTs are located (Hansen *et al*., 2009), with the large conserved globular region at the C-terminus facing the lumen. All GT65R proteins were predicted to be structurally similar to CePOFUT1 (Table S2). The diversity in sequences and similarity in the predicted structure and topology suggest that the GT65R proteins may function as different GTs involved in biosynthesis of various polysaccharides (Hansen *et al*., 2012). The co-expression of several GT65R proteins with GTs involved in secondary cell-wall biosynthesis (Oikawa *et al*., 2010) and the abundance of some other GT65R proteins in Arabidopsis cell suspension cultures (Parsons *et al*., 2012) also provide indirect evidence that GT65R proteins may play a role in primary and secondary cell-wall biosynthesis (Hansen *et al*., 2012).

The characterization of MSR protein demonstrates that biosynthesis of mannan polymers involves more proteins than ManS and galactomannan galactosyl transferase, and raises interesting questions such as how MSR protein affects ManS activity and what other proteins are involved in this process. Future studies aimed at providing answers to these questions will advance the understanding of mannan polysaccharide biosynthetic processes.

## Experimental Procedures

### Plant materials and growth conditions

Fenugreek seeds were purchased from Bulk Foods (http://www.bulkfoods.com/). Seed germination and plant growth were performed as described previously (Alonso *et al*., 2010).

All *AtMSR* T-DNA insertion mutant lines were in the Col-0 background. Seeds of SALK_138965 (*msr1*-*1*), SALK_075245 (*msr1*-*2*), SAIL_576_E11(*msr2*-*2*) and SAIL_46_F12 (*msr2*-*3*) as well as the WT Col-0 were obtained from the Arabidopsis Biological Resource Center (http://abrc.osu.edu/), and GK-017H03 (*msr2*-*1*) seed was obtained from the European Arabidopsis Stock Center (http://arabidopsis.info/). The seeds were sown either directly on soil or plated after surface sterilization onto solid medium containing 1× Murashige and Skoog (MS) salts with vitamins (Caisson Laboratories, http://www.caissonlabs.com), 1% w/v sucrose and 0.8% w/v agar (phytoblend, Caisson Laboratories). After stratification at 4°C for 2–4 days, seeds were germinated in a growth chamber under the same conditions as used by Goubet *et al*. (2009). Two-week-old seedlings germinated on solid medium plates were transferred to soil, and growth continued under the same conditions.

### Laser scanning confocal microscopy

The Golgi marker ST-mRFP (monomeric RFP-tagged transmembrane domain of a rat sialyltransferase) (Renna *et al*., 2005) was provided by Federica Brandizzi at the Department of Energy Plant Research Laboratory, Michigan State University. The Golgi marker and GFP-tagged MSR constructs were transformed into *Agrobacterium* GV3101.

A mixture of agrobacterial cells containing each MSR construct and the Golgi marker were infiltrated into tobacco leaves as described by Davis *et al*. (2010). At 3 days after infiltration, infiltrated leaves were observed under an inverted laser scanning confocal microscope (Olympus FluoView FV1000, http://www.olympus.com/) with a 63× oil immersion objective. GFP signal was detected using an argon laser with a 488 nm excitation wavelength and a 500–545 nm bandpass filter, and mRFP signal was detected with a 559 nm excitation wavelength and a 580–620 nm bandpass filter.

### AIR preparation and sugar composition analysis

Whole primary inflorescence stems were harvested from 6 to 7-week-old plants, lyophilized and ball-milled. Preparation of AIR from stems and removal of starch from the AIR were performed as described by Foster *et al*. (2010). The de-starched AIR was hydrolyzed with trifluoroacetic acid, and the hydrolytic products were subjected to reduction, acetylation and gas chromatography/mass spectrometry analysis as described by Foster *et al*. (2010).

### PACE analysis

The same de-starched AIR samples (500 μg) prepared as described above were incubated with NH_3_ (200 μl, 30 min, 21°C) and lyophilized. Following exhaustive digestion using *Cellvibrio japonicus* mannanase 5A (CjMan5A) and CjMan26A (provided by Professor Harry Gilbert Institute for Cell and Molecular Biosciences, University of Newcastle, UK), samples were derivatized using 2-aminonaphthalene trisulfone (Invitrogen, http://www.invitrogen.com), and separated by polyacrylamide electrophoresis as described previously (Goubet *et al*., 2009). Gels were visualized using a G-box (Syngene, http://www.syngene.com/), fitted with long-wave UV tubes (emitting at 365 nm) and a short-pass detection filter (500–600 nm).

### Immunolabeling of polysaccharides in stem sections

Sections of basal stems from 6-week-old Arabidopsis plants were prepared, pre-treated and blocked as described by Handford *et al*. (2003). Sections were immunolabeled as described by Goubet *et al*. (2009) with minor modifications. Following incubation with primary antibodies raised against mannan (1:100 dilution; Handford *et al*., 2003) and xylan [LM11, 1:100 dilution; McCartney *et al*., 2005; provided by Paul Knox, School of Biology University of Leeds, UK), sections were incubated with the secondary antibodies [Cy3-anti rabbit (mannan) and FITC-labelled anti-rat (xylan), both 1:100 dilution; Jackson ImmunoResearch Laboratories, http://www.jacksonimmuno.com/]. Sections were stained using Calcofluor white (0.1 mg ml^−1^; Sigma, http://www.sigmaaldrich.com), and imaged using an Olympus (http://www.olympus.co.uk/) BX61 microscope. The same exposure was used for each antibody.

### Stem microsome preparation and mannan synthase activity assay

The whole stem was harvested from each 6–7-week-old plant, weighed, and immediately ground in extraction buffer (EB) on ice with a mortar and pestle. EB was prepared as described by Liepman *et al*. (2005), and approximately 1 ml of EB was used per 100 mg of stem. The crude homogenate was centrifuged at 3000 ***g*** for 10 min at 4°C, and the supernatant was centrifuged at 17 000 ***g*** for 20 min at 4°C. The resulting supernatant was then centrifuged at 100 000 ***g*** at 4°C for 90 min to collect microsome membranes. The membrane pellet was resuspended in EB (0.5 μl mg^−1^ stem). Protein concentration was quantified using the BCA protein assay kit (Pierce, http://www.piercenet.com).

The ManS activity assay was performed as described by Liepman *et al*. (2005), with modifications. The assay was performed in a total volume of 40 μl containing 20 μl of freshly prepared microsomes, 21.2 μm cold GDP-Man and 3.8 μm GDP-[^14^C]-Man (9.7 GBq mmol^−1^; PerkinElmer, http://www.perkinelmer.com) at room temperature for 1 h. Reactions were terminated, and products were pelleted and washed as described by Liepman *et al*. (2005). Washed pellets were resuspended in 300 μl of water, and used for liquid scintillation counting as described by Wang *et al*. (2012).

### Additional methods

Additional methods describing the identification of knockout mutants, the generation of double mutants, RNA isolation, RT-PCR, real-time quantitative RT-PCR, gene cloning and construct generation, GUS staining and bioinformatic analysis are described in Data S1. The sequences of primers used for PCR are listed in Table S3.

## SUPPORTING INFORMATION

**Figure S1.** Sequence alignment of TfMSR, AtMSR1 and AtMSR2.

**Figure S2.** Expression of *AtMSR1* in comparison with *AtCSLA* genes.

**Figure S3.** Quantification of PACE band intensity.

**Figure S4.** Quantitative RT-PCR analysis of *MSR* mutants.

**Figure S5.** Phylogenetic tree of TfMSR and the Arabidopsis GT65R proteins.

**Table S1.** Number of *GT65R* genes present in plant genomes.

**Table S2.** Annotated Arabidopsis GT65R proteins.

**Table S3.** Sequences of primers used for PCR.

**Data S1.** Supplementary experimental procedures.

Please note: As a service to our authors and readers, this journal provides supporting information supplied by the authors. Such materials are peer-reviewed and may be re-organized for online delivery, but are not copy-edited or typeset. Technical support issues arising from supporting information (other than missing files) should be addressed to the authors.
