# Supplementary material for: Identification of an additional protein involved in mannan biosynthesis
Source: Plant J. 2012 Oct 19;73(1):105–17. doi: 10.1111/tpj.12019 (PMC3558879; doi:10.1111/tpj.12019)
Supplement: Supplementary file 1 [file tpj0073-0105-SD1.pdf]

```
TfMSR      1  MNSMEIRQAFAGLLTLSMFIMLGNMIKKDHFDY-------PAEEVEIQTTEVSQHDLATV
AtMSR1     1  M-GVDLRQVVAGILTITMFVMLGQMLHRDYFDSLQEKAQGDAQDIEFEGSKVSVKD-GLV
AtMSR2     1  M-GVDLRQVVAGILTITMFVMLGQMLHRDYFDAVQEKVQGDAHDIEFHGSKVAVED-GLV

TfMSR     54  SHISQKSK----QNDKALKPCWNPPTLKEVEQSKGFIIFSLTNGPEYHIAQVADAVVVAK
AtMSR1    59  GTVE-GSKGLWMEDNTDLTPCWPTLLSDDAVSSKGYVTFSLTNGPEYHISQITDAVMVAK
AtMSR2    59  RAFEAGTKGPWMEDSHELKPCWSISQSDEAVSSKGYVTFSLTNGPEYHVSQITDAVMVAK

TfMSR    110  YLGATLVLPDIKNSKSGNSMNLGDIYDVENVLNKLNGLVKVTKTLPPHVSTRNTPIVRVP
AtMSR1   118  HLGATLVLPDIRGSKPGDERNFEDIYDADKLIKSLENVVKVVKKLPEEVSLRNMAIVKVP
AtMSR2   119  HLGATLVLPDIRGSKPGDEMKFEDIYDVDKLIKTLESVVKVVRKLPSHVSLRDIAIVKVP

TfMSR    170  NKVSQDYIMKKLKPIYQAKGIIKIESYFPSKN-TISRNNNSLESLLCQTMFGGTLELKKE
AtMSR1   178  TRVTEDYIKEHIDPIFKSKGNIRVASYFPSVNLRKSSQDGETDPVACLAMF-GSLELQPE
AtMSR2   179  TRVAEDYIKEHIDPIFKSKGNIRVTTYFPSVNLRKSSQGAETDPVSCLAMF-GSLELQPA

TfMSR    229  IQEEAESIVQKLETWSQESNGPFVAVDLRIEGL-KNECNGKDGKGRKQCYQGHEIGEFLK
AtMSR1   237  VNAVAESMVERLRTHSRKSGGRFIAVDLRIDILEKKNCHTTGVVGSKTCYNAQEIALFLR
AtMSR2   238  VNELVESMIQRLKTHSKKSGGRFIAIDLRVEILEKKNCHETGAVGSKTCYNAQEIALFLR

TfMSR    288  RIGFGQETVIYVTQTKWSPDLNSLRYMFPKTYTKENIMSSTKKEKFINSESIEFEKAIDF
AtMSR1   297  KLGFASDTTIYLTQPRWDSSLNILKDIFPKTFTKEAIMPASKRSKYLESVSSEYENVIDF
AtMSR2   298  KLGFDSDTTIYLTQPRWESSLNILKDIFPKTYTKEAIMPSDKKTKYLELENSEYENVIDF

TfMSR    348  YICSESDVFVPSILGPFYENVAGMRIVSGKNEIIVPSEVVSPSASASEHMSPYVTKKNHL
AtMSR1   357  YISSRSDVFVPAISGLFYANTVGKRIALGKPQVLVPAEISETSGLATDFISPYISKKNHL
AtMSR2   358  YISSRSDVFVPAIPGLFYANTVGKRIALGKPQVLVPAEISGTSGLPANYISPYISKKNHL

TfMSR    408  AYKCFC
AtMSR1   417  AYSCFC
AtMSR2   418  AYSCFC
```
