# Supplementary figures and images for: Identification of an additional protein involved in mannan biosynthesis
Source: Plant J. 2012 Oct 19;73(1):105–17. doi: 10.1111/tpj.12019 (PMC3558879; doi:10.1111/tpj.12019)

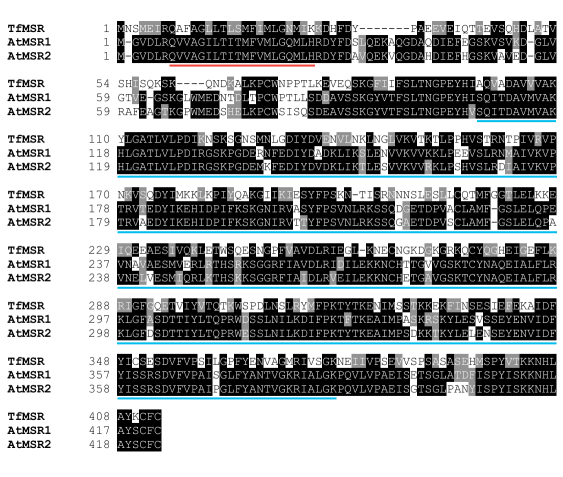

Supplement: Supplementary file 2 [file tpj0073-0105-SD9.png]

**(a)**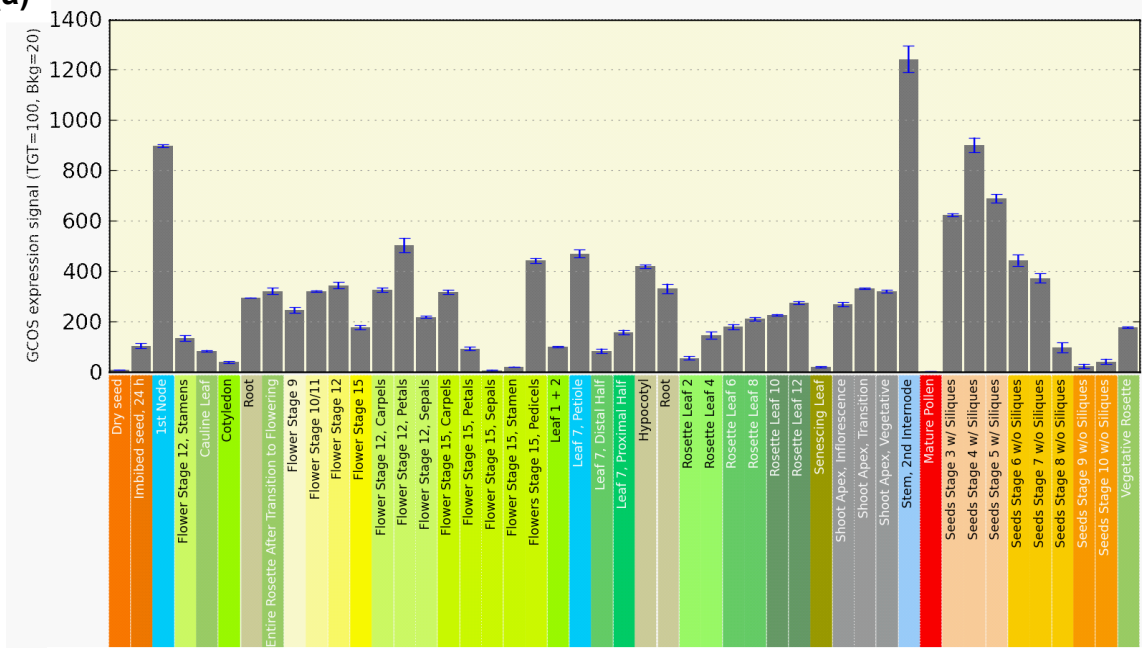**(b)**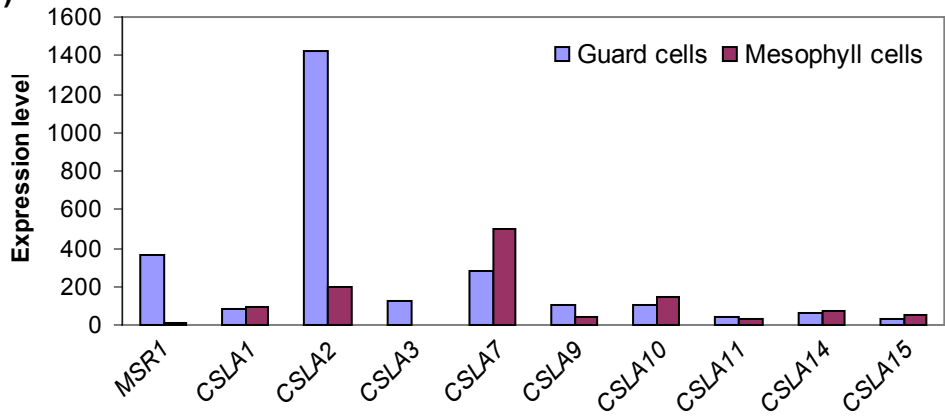**(c)**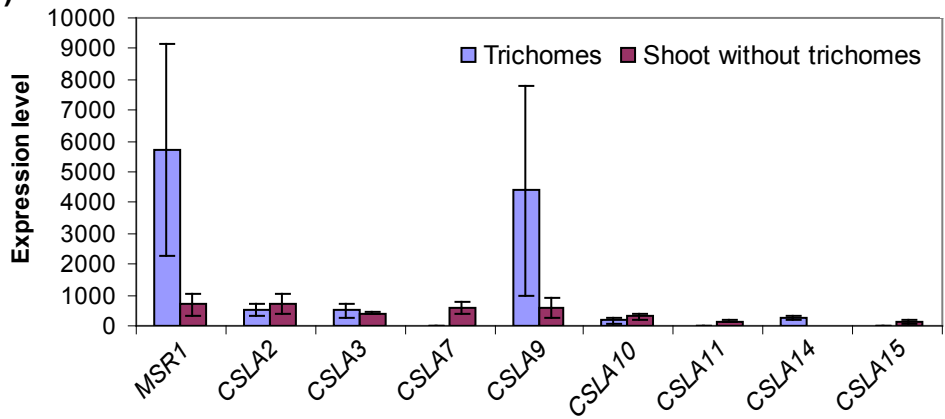**(d)**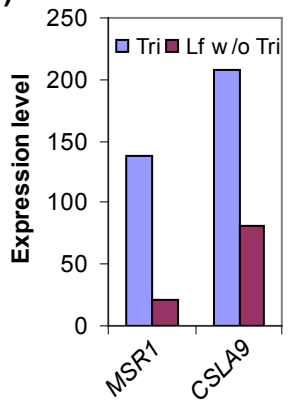

Supplement: Supplementary file 4 [file tpj0073-0105-SD2.pdf]

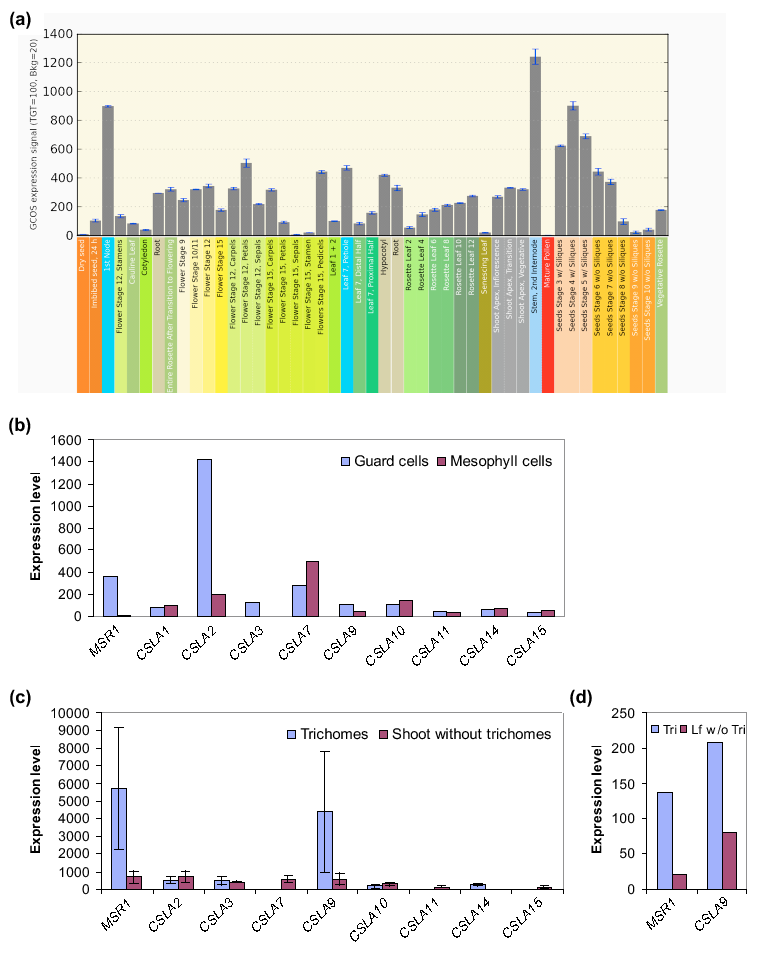

Supplement: Supplementary file 5 [file tpj0073-0105-SD10.png]

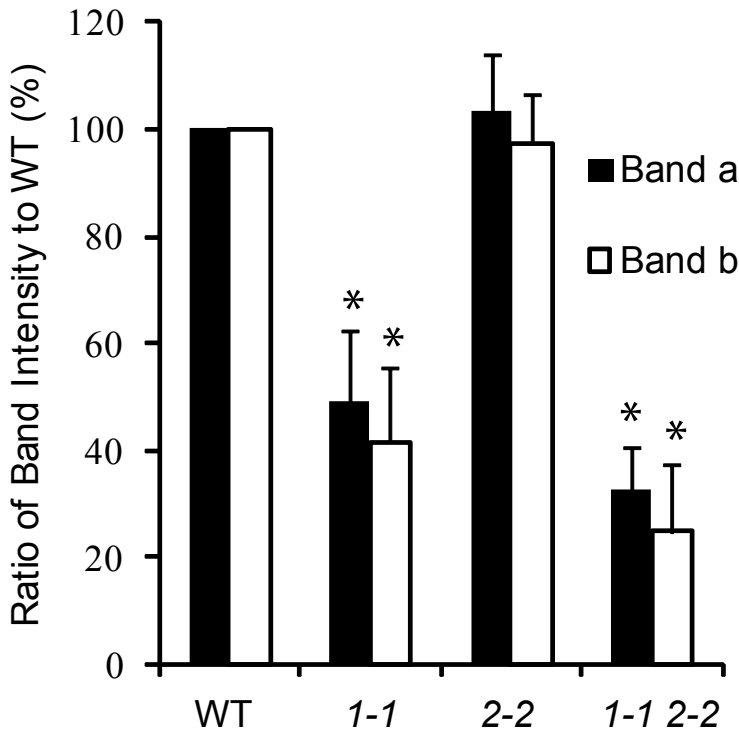

Supplement: Supplementary file 7 [file tpj0073-0105-SD3.pdf]

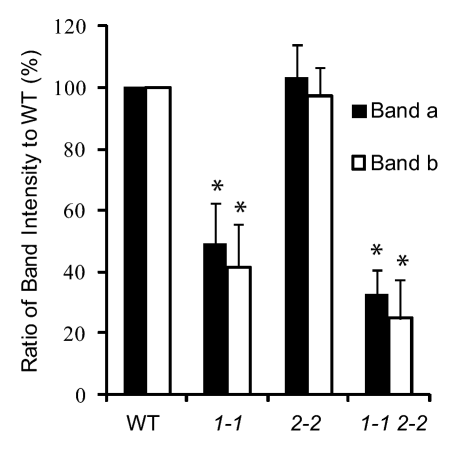

Supplement: Supplementary file 8 [file tpj0073-0105-SD11.png]

**(a)**

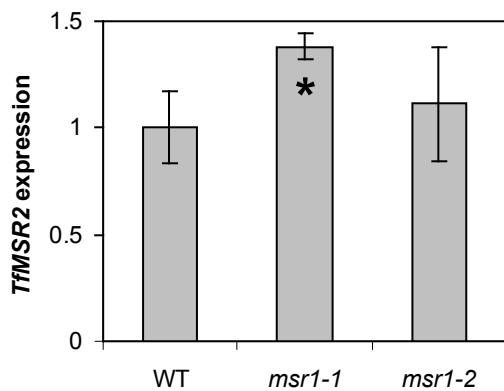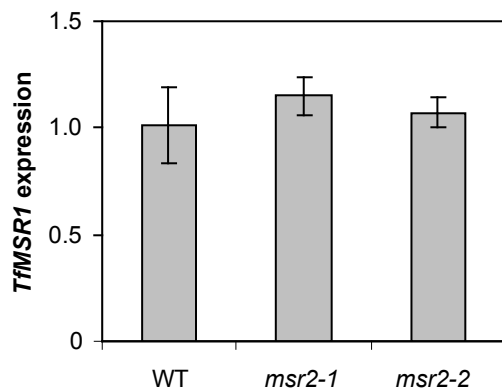

**(b)**

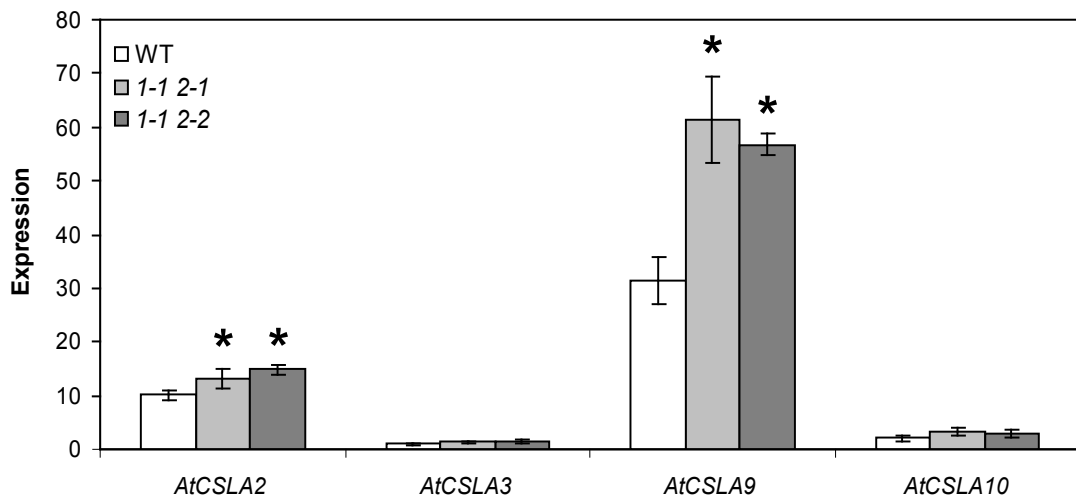

Supplement: Supplementary file 10 [file tpj0073-0105-SD4.pdf]

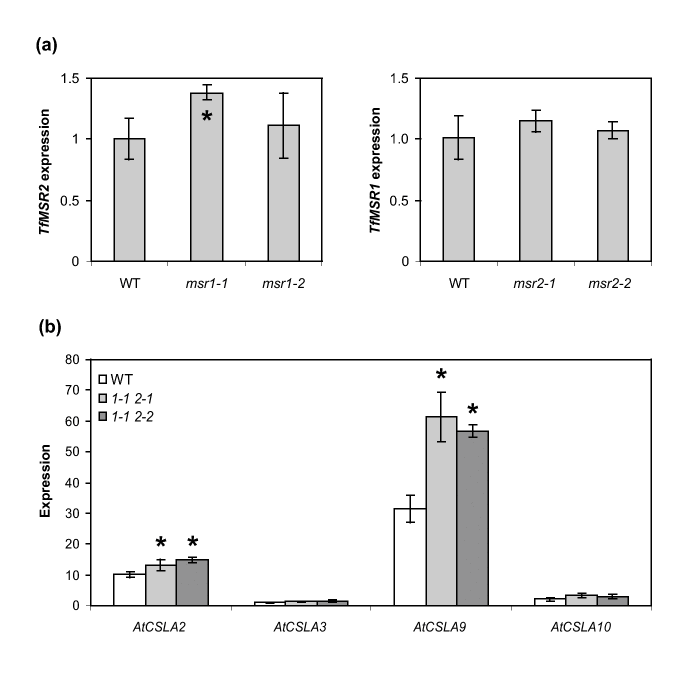

Supplement: Supplementary file 11 [file tpj0073-0105-SD12.png]

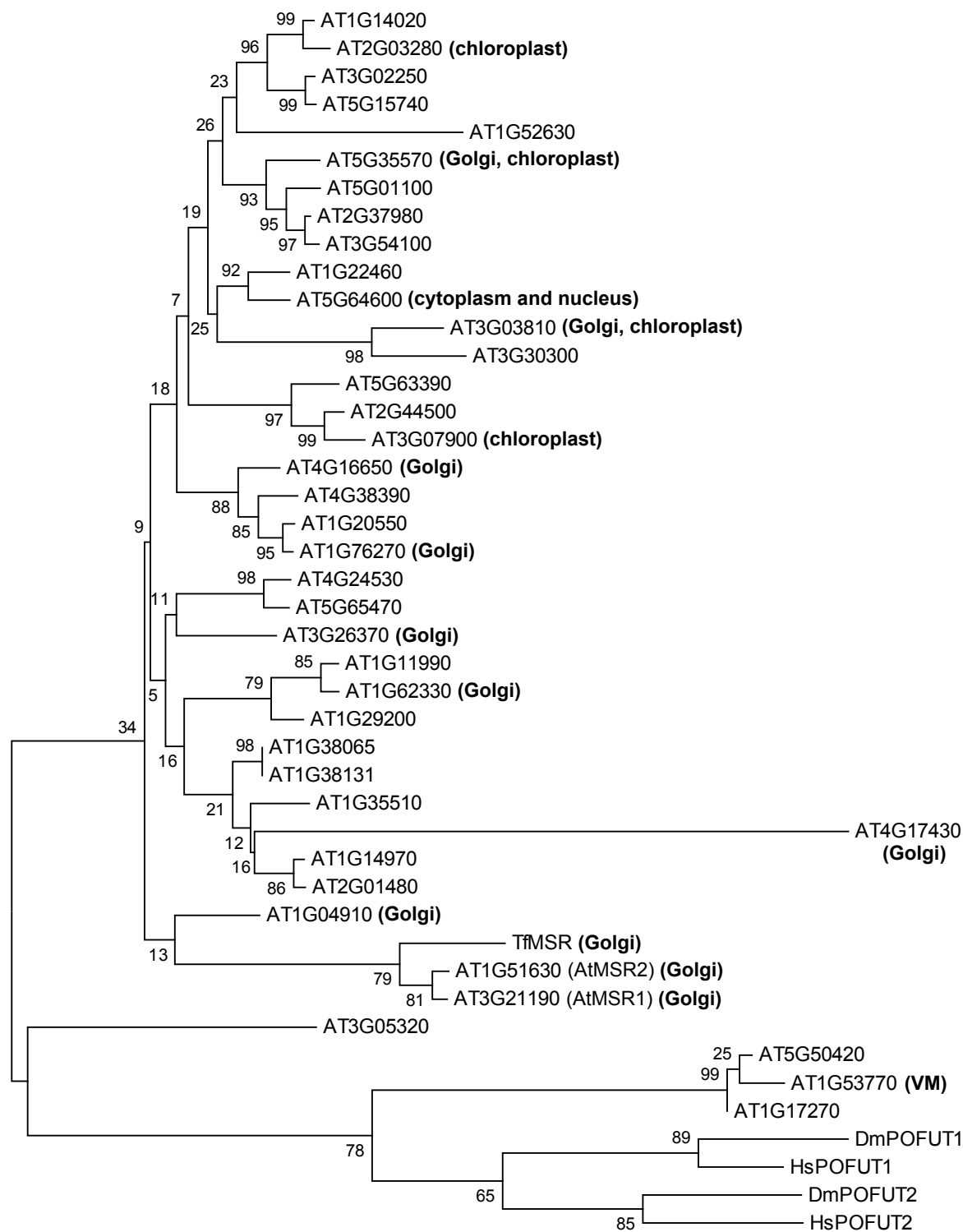

0.5

Supplement: Supplementary file 13 [file tpj0073-0105-SD5.pdf]

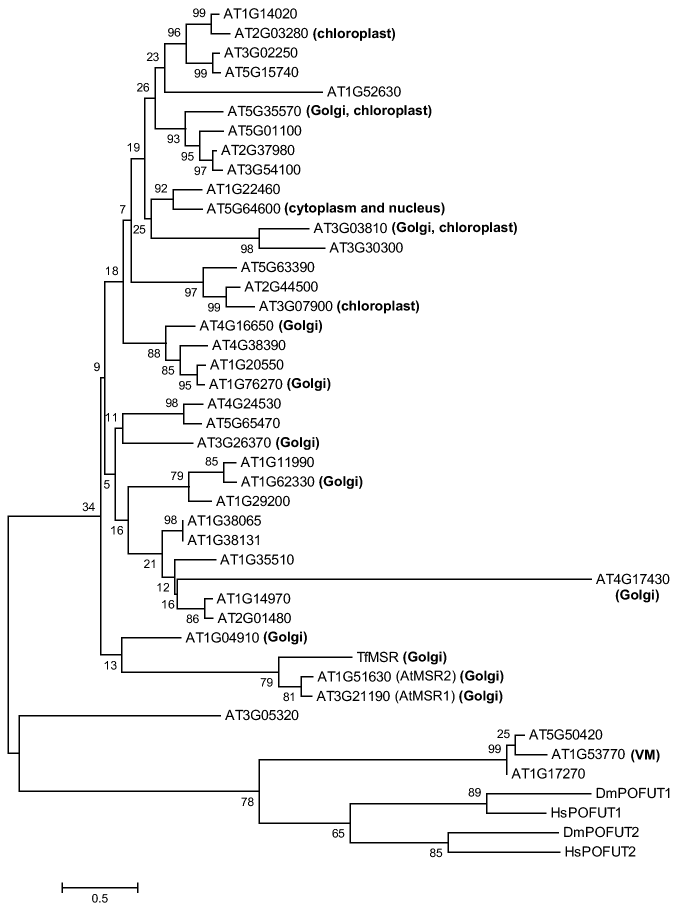

Supplement: Supplementary file 14 [file tpj0073-0105-SD13.png]
