# Supplementary material for: Identification of an additional protein involved in mannan biosynthesis
Source: Plant J. 2012 Oct 19;73(1):105–17. doi: 10.1111/tpj.12019 (PMC3558879; doi:10.1111/tpj.12019)
Supplement: Supplementary file 16 [file tpj0073-0105-SD6.doc]

**Supplemental Tables**

**Table S1** Number of *GT65R* genes present in plant genomes

| Species | Clade | Reference | *GT65R* |
| --- | --- | --- | --- |
| *Micromonas pusilla CCMP1545* | green algae (unicellular) | Worden *et al*., 2009 | 0 |
| *Micromonas sp. RCC299* | green algae (unicellular) | Worden *et al*., 2009 | 0 |
| *Ostreococcus lucimarinus* | green algae (unicellular) | Palenik *et al*., 2007 | 0 |
| *Ostreococcus tauri* | green algae (unicellular) | Derelle *et al*., 2006 | 0 |
| *Chlamydomonas reinhardtii* | green algae (unicellular) | Merchant *et al*., 2007 | 0 |
| *Volvox carteri* | green algae (multicellular) | Prochnik *et al*., 2010 | 0 |
| *Physcomitrella patens* | moss | Rensing *et al*., 2008 | 25 |
| *Arabidopsis thaliana* | dicot | AGI, 2000 | 39 |
| *Glycine max* | dicot | Schmutz *et al*., 2010 | 73 |
| *Populus trichocarpa* | dicot | Tuskan *et al*., 2006 | 46 |
| *Brachypodium distachyon* | monocot | IBI, 2010 | 29 |
| *Oryza sativa* | monocot | Goff *et al*., 2002; Yu *et al*., 2002 | 30 |

AGI, Arabidopsis Genome Initiative; IBI, International Brachypodium Initiative.

**Table S2** Annotated *Arabidopsis* GT65R proteins

| **Gene designation** | **Predicted**  **localization^a^** | **Reported**  **localization^b^** | **Predicted**  **TMD^c^** | **Structure**  **homologue^d^** | **Expression pattern^e^** | **Expression threshold^e^** |
| --- | --- | --- | --- | --- | --- | --- |
| At1g04910 | M (0.781) | Golgi^1,2,3^, PM^4^ | 1, 1 | CePOFUT1 (10.90) | all tissues | 172.11 |
| At1g11990 | O (0.965) |  | 1, 1 | CePOFUT1 (12.84) | pollen | 1015.08 |
| At1g14020 | O (0.919) |  | 0, 1 | CePOFUT1 (12.18) | mainly pollen | 219.63 |
| At1g14970 | C (0.645) |  | 1, 1 | CePOFUT1 (15.16) | mainly pollen | 325.13 |
| At1g17270 | O (0.912) |  | 1, 1 | CePOFUT1 (30.16) | no data | no data |
| At1g20550 | M (0.910) |  | 1, 1 | CePOFUT1 (11.39) | silique/seed | 102.9 |
| At1g22460 | M (0.789) |  | 1, 1 | CePOFUT1 (12.64) | pollen/stamen | 432.56 |
| At1g29200 | O (0.492) |  | 0, 1 | CePOFUT1 (11.04) | pollen/stamen, stem | 79.06 |
| At1g35510 | C (0.764) |  | 1, 2 | CePOFUT1 (12.19) | all tissues | 106.11 |
| At1g38065 | C (0.541) |  | 0, 1 | CePOFUT1 (15.62) | All tissues | 131.61 |
| At1g38131 | C (0.942) |  | 1, 2 | CePOFUT1 (13.96) | All tissues | 131.61 |
| At1g51630 | S (0.912) | Golgi^1,2,3^, CW^5,6^ | 1, 1 | CePOFUT1 (11.95) | no data | no data |
| At1g52630 | S (0.987) |  | 1, 1 | CePOFUT1 (12.76) | mainly seed | 54.98 |
| At1g53770 | O (0.805) | VM^7^ | 1,1 | CePOFUT1 (23.28) | all tissues | 78.05 |
| At1g62330 | O (0.863) | Golgi^3^ | 1, 1 | CePOFUT1 (10.04) | all tissues | 138.47 |
| At1g76270 | M (0.876) | Golgi^2^ | 1, 1 | CePOFUT1 (11.34) | all tissues | 40.63 |
| At2g01480 | C (0.888) |  | 1, 1 | CePOFUT1 (11.84) | pollen, dry seed | 175.23 |
| At2g03280 | O (0.969) | Chloroplast^8^ | 1, 1 | CePOFUT1 (14.18) | seed | 179.01 |
| At2g37980 | C (0.888) |  | 2, 2 | CePOFUT1 (10.39) | pollen | 1527.76 |
| At2g44500 | C (0.643) |  | 1, 2 | CePOFUT1 (11.22) | most tissues | 668.81 |
| At3g02250 | O (0.931) |  | 1, 1 | CePOFUT1 (14.77) | most tissues | 191.56 |
| At3g03810 | S (0.967) | Golgi^3^, Chloroplast^8^ | 1, 1 | CePOFUT1 (9.55) | all tissues | 96.75 |
| At3g05320 | M (0.351) |  | 1, 1 | CePOFUT1 (11.74) | most tissues | 180.33 |
| At3g07900 | C (0.341) | Chloroplast^9^ | 1, 2 | CePOFUT1 (10.50) | root, seed^10^ | 26.33 |
| At3g21190 | S (0.896) | Golgi^1,2,3^, PM^4^ | 1, 1 | CePOFUT1 (12.05) | most tissues | 1242.15 |
| At3g26370 | M (0.335) | Golgi^1,3^ | 1, 1 | CePOFUT1 (13.44) | all tissues | 192.16 |
| At3g30300 | S (0.825) |  | 1, 1 | CePOFUT1 (9.37) | most tissues | 207.71 |
| At3g54100 | O (0.543) |  | 1, 1 | CePOFUT1 (9.72) | all tissues | 134.08 |
| At4g16650 | M (0.496) | Golgi^1,3^ | 1, 1 | CePOFUT1 (14.78) | all tissues | 130.56 |
| At4g17430 | O (0.815) | Golgi^1,3^ | 1, 1 | CePOFUT1 (20.27) | most tissues | 77.18 |
| At4g24530 | M (0.315) |  | 0, 1 | CePOFUT1 (12.39) | all tissues | 100.88 |
| At4g38390 | O (0.882) |  | 1, 2 | CePOFUT1 (14.21) | pollen/stamen | 105.34 |
| At5g01100 | C (0.606) |  | 1, 1 | CePOFUT1 (10.91) | no data | no data |
| At5g15740 | O (0.913) |  | 1, 1 | CePOFUT1 (12.06) | mainly seed/silique | 1210.46 |
| At5g35570 | C (0.772) | Golgi^3^, Chloroplast^8^ | 1, 1 | CePOFUT1 (9.53) | most tissues | 62.84 |
| At5g50420 | O (0.953) |  | 1, 1 | CePOFUT1 (25.78) | most tissues | 129.53 |
| At5g63390 | C (0.569) |  | 1, 2 | CePOFUT1 (14.56) | undetectable^11^ | 3.06 |
| At5g64600 | M (0.842) | Cyt and Nuc^10^ | 1, 1 | CePOFUT1 (13.55) | stamen/pollen | 87.83 |
| At5g65470 | S (0.323) |  | 0, 0 | CePOFUT1 (14.78) | mainly dry seed | 1255.96 |

^a^Cellular localization was predicted by TargetP software (Emanuelss*on et a*l., 2007). The value in parentheses indicates a prediction score. C, chloroplasts; M, mitochondria; S, protein with a signal peptide localized to the secretory pathway (endomembrane sysyem); O, localization other than chloroplasts, mitochondria and the secretory pathway;

^b^Localization determined by experiments. CW, cell wall; Cyt, cytoplasm; Nuc, nucleus; PM, plasma membrane; VM: vacuolar membrane. References: ^1^Dunkley *et al*., 2004; ^2^Dunkley *et al*., 2006; ^3^Parsons *et al*., 2012; ^4^Mitra *et al*., 2009; ^5^Kwon *et al*., 2005; ^6^Bayer *et al*., 2006; ^7^Jaquinod *et al*., 2007; ^8^Zybailov *et al*., 2008; ^9^Kleffmann *et al*., 2004; ^10^Koroleva *et al*., 2005.

^c^Number of transmembrane domains predicted by TMHMM2.0 (http://www.cbs.dtu.dk/services/TMHMM/) and ARAMEMNON (http://aramemnon.botanik.uni-koeln.de/), respectively. The cut-off value for ARAMEMNON prediction is 0.50. With the exceptions of At1g14020, At1g29200, At1g38065, At4g24530 and At5g65470, all GT65R proteins were predicted by TMHMM2.0 to have a large C-terminal globular region located at the side opposite to the cytosol.

^d^Distant structure homologue predicted by the FUGUE program (S*hi et a*l., 2001). The value in the parentheses shows a ZSCORE, which indicates 99% prediction confidence if ZSCORE ≥ the recommended cut-off value 6.0.

^e^Based on publically available AtGenExpress microarray data (Schm*id et a*l., 2005) visualized by the Arabidopsis eFP Browser (Wint*er et a*l., 2007). ^10,11^Near or lower than the background level 20.

Five genes (*At1g29200, At1g52630, At1g53770, At2g03280, At2g44500*) have more than one gene model. For these genes, only the first model is shown.

**Table S3** Sequences of primers used for PCR

| Primer | Sequence |
| --- | --- |
| At1g51630-cDNA-953F | 5’-GCGGTTGAAGACACATAGCA-3’ |
| At1g51630-cDNA-1394R | 5’-GGCTGGAACGAGAACTTGAG-3’ |
| At1g51630-5’UTR-34F | 5’-TTGACAAAATCATCAAAAGGGA-3’ |
| At1g51630-CDS-273R | 5’-TGACGATACCGCTTCATCAG-3’ |
| At1g51630-CDS-262F | 5’-GCGGTATCGTCAAAGGGTTA-3’ |
| At1g51630-CDS-882R | 5’-CAATGCAATCTCTTGTGCGT-3’ |
| At1g51630-GABI-LP | 5’-GACTATCTTCCATCCAAGGCC-3’ |
| At1g51630-GABI-RP | 5’-CCTTGACGACAAATCAATTAAGG-3’ |
| At1g51630-Prom-1998F | 5’-CACCAAAAGCATTTCGTTTTCCCA-3’ |
| At1g51630-Prom-56R | 5’-GAAATGGGTTTTTCCAAGGG-3’ |
| At1g51630+Start | 5’-CACCATAATGGGTGTGGATTTGAGGC-3’ |
| At1g51630+Stop | 5’-TCAGCAAAAGCATGAATACGC-3’ |
| At1g51630-Stop | 5’-GCAAAAGCATGAATACGCCA-3’ |
| AT3G21190-CDS-258F | 5’-CGCTGTATCATCGAAAGGGT-3’ |
| AT3G21190-CDS-725R | 5’-TCAGCCACTGCATTCACTTC-3’ |
| At3g21190-Prom-1480F | 5’-CACCGGAGGATCGGTACGGT-3’ |
| At3g21190-Prom-1R | 5’-TTCTTACCTTTCTCTCTAACAAAAAAAAACAGA-3’ |
| At3g21190-SALK-LP | 5’-CAAGACCTTCCATTTTTGGATC-3’ |
| At3g21190-SALK-RP | 5’-TACAGGATCAGTTTCGCCATC-3’ |
| At3g21190+Start | 5’-CACCATGGGTGTTGATTTGAGGCA-3’ |
| At3g21190+Stop | 5’-TCAGCAAAAGCATGAATAAGCC-3’ |
| At3g21190-Stop | 5’-GCAAAAGCATGAATAAGCCAAAT-3’ |
| AT4G26410-Fwd | 5’-GAGCTGAAGTGGCTTCCATGAC-3’ |
| AT4G26410-Rev | 5’-GGTCCGACATACCCATGATCC-3’ |
| AtCslA2-CDS-1156F | 5’-GCGTTGTTCTTCCTCTCACAATT-3’ |
| AtCslA2-CDS-1214R | 5’-CGAACCCCAAATCGGAACT-3’ |
| AtCslA3-CDS-1366F | 5’-CATCGCACAAAAGCGACATT-3’ |
| AtCslA3-CDS-1433R | 5’-TCAGTAACAACCCATTCGTTAACC-3’ |
| AtCslA9-CDS-1483F | 5’-GTGGGATGTTATGACGCGTTT-3’ |
| AtCslA9-CDS-1550R | 5’-GCGATTGCTTGTGCGAAA-3’ |
| AtCslA10-CDS-1370F | 5’-TGCACCGGACAAAAGGAACT-3’ |
| AtCslA10-CDS-1429R | 5’-CCCATTCGTTCACTCTTTGTCTT-3’ |
| AtMSR1-CDS-304F | 5’-CCTGAGTACCATATCTCTCAGATCAC-3' |
| AtMSR1-CDS-448R | 5’-TAATCAGCTTATCCGCATCATAGATA-3' |
| AtMSR2-CDS-532F | 5’-CCTACCCGAGTTGCAGAAGA-3' |
| AtMSR2-CDS-673R | 5’-AAGAGACAGGGTCGGTTTCA-3' |
| EIF4A-CDS-55F | 5’-AAACTCAATGAAGTACTTGAGGGACA-3’ |
| EIF4A-CDS-181R | 5’-TCTCAAAACCATAAGCATAAATACCC-3’ |
| o8409 | 5’-ATATTGACCATCATACTCATTGC-3’ |
| SAIL_576_E11-LP | 5’-TCTGGTGTTTCAGATGAAGCG-3’ |
| SAIL_576_E11-RP | 5’-GCAAATGGGTGAAGACTTCAG-3’ |
| SAIL_46_F12-LP | 5’-CATTTTGGTTTCGGTGAATTG-3’ |
| SAIL_46_F12-LP | 5’-AAAGCATGAATACGCCAAGTG-3’ |
| SAIL-LB3 | 5’-TAGCATCTGAATTTCATAACCAATCTCGATACAC-3’ |
| LBa1 | 5’-TGGTTCACGTAGTGGGCCATCG-3’ |
| TfManS-17F | 5’-CAGTTAGGGCAAGCCTGAAG-3’ |
| TfManS-450R | 5’-TGCCTTAGTTCGATGCAGTG-3’ |
| TfMSR-CDS-728F | 5’-GGAGCCAAGAATCAAATGGA-3’ |
| TfMSR-CDS-1114R | 5’-TCATCCCAGCAACATTTTCA-3’ |
| TfMSR+Start | 5’-CACCATGAATTCAATGGAAATTAGGCAA-3’ |
| TfMSR+Stop | 5’-TCAACAAAAGCATTTATAAGCCAA-3’ |
| TfMSR-Stop | 5’-ACAAAAGCATTTATAAGCCAAATGG-3’ |
| TfEF1a-CDS-324F | 5’-GGCTGATTGTGCTGTTCTCA-3’ |
| TfEF1a-CDS-707R | 5’-AGTCTGAGGGGCTTGTCTGA-3’ |

**Arabidopsis Genome Initiative**. (2000) Analysis of the genome sequence of the flowering plant *Arabidopsis thaliana*. *Nature*, **408**, 796-815.

**Bayer, E.M., Bottrill, A.R., Walshaw, J., Vigouroux, M., Naldrett, M.J., Thomas, C.L. and Maule, A.J.** (2006) *Arabidopsis* cell wall proteome defined using multidimensional protein identification technology. *Proteomics*, **6**, 301-311.

**Derelle, E., Ferraz, C., Rombauts, S. *et al.*** (2006) Genome analysis of the smallest free-living eukaryote *Ostreococcus tauri* unveils many unique features. *Proc. Natl. Acad. Sci. USA*, **103**, 11647-11652.

**Dunkley, T.P., Hester, S., Shadforth, I.P. *et al.*** (2006) Mapping the *Arabidopsis* organelle proteome. *Proc. Natl. Acad. Sci. USA*, **103**, 6518-6523.

**Dunkley, T.P., Watson, R., Griffin, J.L., Dupree, P. and Lilley, K.S.** (2004) Localization of organelle proteins by isotope tagging (LOPIT). *Mol. Cell. Proteomics*, **3**, 1128-1134.

**Emanuelsson, O., Brunak, S., von Heijne, G. and Nielsen, H.** (2007) Locating proteins in the cell using TargetP, SignalP and related tools. *Nat. Protoc*. **2**, 953-971.

**Goff, S.A., Ricke, D., Lan, T.H. *et al.*** (2002) A draft sequence of the rice genome (*Oryza sativa* L. ssp. japonica). *Science*, **296**, 92-100.

**International Brachypodium Initiative.** (2010) Genome sequencing and analysis of the model grass *Brachypodium distachyon*. *Nature*, **463**, 763-768.

**Jaquinod, M., Villiers, F., Kieffer-Jaquinod, S., Hugouvieux, V., Bruley, C., Garin, J. and Bourguignon, J.** (2007) A proteomics dissection of *Arabidopsis thaliana* vacuoles isolated from cell culture. *Mol. Cell. Proteomics*, **6**, 394-412.

**Kleffmann, T., Russenberger, D., von Zychlinski, A., Christopher, W., Sjolander, K., Gruissem, W. and Baginsky, S.** (2004) The *Arabidopsis thaliana* chloroplast proteome reveals pathway abundance and novel protein functions. *Curr. Biol*. **14**, 354-362.

**Koroleva, O.A., Tomlinson, M.L., Leader, D., Shaw, P. and Doonan, J.H.** (2005) High-throughput protein localization in *Arabidopsis* using Agrobacterium-mediated transient expression of GFP-ORF fusions. *Plant J*. **41**, 162-174.

**Kwon, H.K., Yokoyama, R. and Nishitani, K.** (2005) A proteomic approach to apoplastic proteins involved in cell wall regeneration in protoplasts of *Arabidopsis* suspension-cultured cells. *Plant Cell Physiol*. **46**, 843-857.

**Merchant, S.S., Prochnik, S.E., Vallon, O. *et al.*** (2007) The *Chlamydomonas* genome reveals the evolution of key animal and plant functions. *Science*, **318**, 245-250.

**Mitra, S.K., Walters, B.T., Clouse, S.D. and Goshe, M.B.** (2009) An efficient organic solvent based extraction method for the proteomic analysis of *Arabidopsis* plasma membranes. *J. Proteome Res*. **8**, 2752-2767.

**Palenik, B., Grimwood, J., Aerts, A. *et al.*** (2007) The tiny eukaryote *Ostreococcus* provides genomic insights into the paradox of plankton speciation. *Proc. Natl. Acad. Sci. USA*, **104**, 7705-7710.

**Parsons, H.T., Christiansen, K., Knierim, B., Carroll, A., Ito, J., Batth, T.S., Smith-Moritz, A.M., Morrison, S., McInerney, P., Hadi, M.Z., Auer, M., Mukhopadhyay, A., Petzold, C.J., Scheller, H.V., Loqué, D. and Heazlewood, J.L.** (2012) Isolation and proteomic characterization of the *Arabidopsis* Golgi defines functional and novel components involved in plant cell wall biosynthesis. *Plant Physiol*. **159**, 12-26.

**Prochnik, S.E., Umen, J., Nedelcu, A.M. *et al.*** (2010) Genomic analysis of organismal complexity in the multicellular green alga *Volvox carteri*. *Science*, **329**, 223-226.

**Rensing, S.A., Lang, D., Zimmer, A.D. *et al.*** (2008) The *Physcomitrella* genome reveals evolutionary insights into the conquest of land by plants. *Science*, **319**, 64-69.

**Schmid, M., Davison, T.S., Henz, S.R., Pape, U.J., Demar, M., Vingron, M., Scholkopf, B., Weigel, D. and Lohmann, J.U.** (2005) A gene expression map of *Arabidopsis thaliana* development. *Nat. Genet*. **37**, 501-506.

**Schmutz, J., Cannon, S.B., Schlueter, J. *et al.*** (2010) Genome sequence of the palaeopolyploid soybean. *Nature*, **463**, 178-183.

**Shi, J.Y., Blundell, T.L. and Mizuguchi, K.** (2001) FUGUE: Sequence-structure homology recognition using environment-specific substitution tables and structure-dependent gap penalties. *J. Mol. Biol*. **310**, 243-257.

**Tuskan, G.A., Difazio, S., Jansson, S. *et al.*** (2006) The genome of black cottonwood, Populus trichocarpa (Torr. & Gray). *Science*, **313**, 1596-1604.

**Winter, D., Vinegar, B., Nahal, H., Ammar, R., Wilson, G.V. and Provart, N.J.** (2007) An "Electronic Fluorescent Pictograph" browser for exploring and analyzing large-scale biological data sets. *PLoS One*, **2**, e718.

**Worden, A.Z., Lee, J.H., Mock, T. *et al.*** (2009) Green evolution and dynamic adaptations revealed by genomes of the marine picoeukaryotes *Micromonas*. *Science*, **324**, 268-272.

**Yu, J., Hu, S., Wang, J. *et al.*** (2002) A draft sequence of the rice genome (*Oryza sativa* L. ssp. indica). *Science*, **296**, 79-92.

**Zybailov, B., Rutschow, H., Friso, G., Rudella, A., Emanuelsson, O., Sun, Q. and van Wijk, K.J.** (2008) Sorting signals, N-terminal modifications and abundance of the chloroplast proteome. *PLoS One*, **3**, e1994.
