# Supplementary material for: Identification of an additional protein involved in mannan biosynthesis
Source: Plant J. 2012 Oct 19;73(1):105–17. doi: 10.1111/tpj.12019 (PMC3558879; doi:10.1111/tpj.12019)
Supplement: Supplementary file 17 [file tpj0073-0105-SD7.doc]

**Methods S1.** Supplementary experimental procedures

**Identification of knockout mutants and generation of double mutants**

Genomic DNA was isolated from leaves using the DNeasy Plant Mini Kit (QiaGen, http://www.qiagen.com). Each T-DNA insertion line was genotyped by polymerase chain reaction (PCR) with genomic DNA as a template. The following primer pairs were used: At3G21190-SALK-LP / At3G21190-SALK-RP and At3G21190-SALK-RP / LBa1 (left border primer for the SALK T-DNA) for *msr1-1* and *msr1-2*; At1g51630-GABI-LP / At1g51630-GABI-RP and At1g51630-GABI-RP / o8409 (left border primer for the GABI-KAT T-DNA) for *msr2-1*; SAIL_576_E11-LP / SAIL_576_E11-RP and SAIL_576_E11-RP / SAIL-LB3 (left border primer for the SAIL T-DNA) for *msr2-2*; and SAIL_46_F12-LP / SAIL_46_F12-RP and SAIL_46_F12-RP / SAIL-LB3 for *msr2-3*. The sequences of primers used in this work are all listed in Table S3. The location of the T-DNA insert within the genes was verified by sequencing T-DNA-tagged PCR products amplified by a T-DNA left border primer and a gene-specific primer. True transcriptional knockouts were identified by RT-PCR as described below. To generate double mutants, homozygous *msr1-1* plants were crossed to homozygous *msr2-1* or *msr2-2* plants. The homozygous *msr1-1 msr2-1* and *msr1-1 msr2-2* double mutants were identified by PCR using the same primer pairs as for each single mutant.

**RNA isolation and RT-PCR**

For RT-PCR analysis of *TfMSR*, RNA was isolated from leaves, embryos and endosperms of fenugreek as described by Wang *et al*. (2012). RNA was treated with DNase I and purified using the QiaGen RNeasy Plant Mini Kit (http://www.qiagen.com/). The first-strand cDNA was synthesized using SuperScript III Reverse Transcriptase from Invitrogen (http://www.invitrogen.com). PCR was performed using the GoTaq Green Master Mix (Promega, http://www.promega.com/). The primers used were: TfMSR-CDS-728F and TfMSR-CDS-1114R for *TfMSR*, TfManS-17F and TfManS-450R for *TfManS*, and TfEF1a-CDS-324F and TfEF1a-CDS-707R for *TfEF-1-α* (control).

For RT-PCR of two *AtMSAR* genes to examine their expression in WT *Arabidopsis* plants, RNA was isolated from Col-0 seedlings or different tissues using the TRIZOL Reagent (Invitrogen). DNase I treatment and purification of RNA, first-strand cDNA synthesis, and PCR were performed as described above. The primers used were: AT3G21190-CDS-258F and AT3G21190-CDS-725R for *AtMSR1*, At1g51630-cDNA-953F and At1g51630-cDNA-1394R for *AtMSR2*, and EIF4A-CDS-55F and EIF4A-CDS-181R for the *Arabidopsis* translation initiation factor 4A-1 gene (*AtEIF4A1*, *At3g13920*) as a control. The primers for *AtMSR1* or *AtMSR2* were located in two different exons, and the amplified genomic DNA fragment of Col-0 was thus larger than the cDNA fragment. The two primers for *AtEIF4A1* cDNA were designed in a way that only a cDNA fragment could be amplified by PCR.

For RT-PCR of *AtMSR* genes to identify true knockout mutants, RNA was isolated from primary stems of the WT and *msr* mutant plants. The primers used for RT-PCR are: AT3G21190-CDS-258F and AT3G21190-CDS-725R for *AtMSR1* in *msr1-1* and *msr1-2* lines; AT1G51630-5’UTR-34F and AT1G51630-CDS-273R for *AtMSR2* in *msr2-1*; At1g51630-CDS-262F and At1g51630-CDS-882R for *AtMSR2* in *msr2-2*; At1g51630-cDNA-953F and At1g51630-cDNA-1394R for *AtMSR2* in *msr2-3*; and EIF4A-CDS-55F and EIF4A-CDS-181R for *AtEIF4A1* as a control.

# Real-time quantitative RT-PCR

RNA was isolated from primary stems of the WT and *msr* mutant plants, and was used to synthesize first-strand cDNA as aforementioned. The relative expression of *AtMSR* and *AtCSLA* genes was analyzed by real-time RT-PCR using the Power SYBR Green PCR Master Mix (Applied Biosystems, www.appliedbiosystems.com) with the first-strand cDNA as a template. Primers used for PCR were: AtMSR1-CDS-304F and AtMSR1-CDS-448R for *AtMSR1*, AtMSR2-CDS-532F and AtMSR2-CDS-673R for *AtMSR2*, AtCslA2-CDS-1156F and AtCslA2-CDS-1214R for *AtCSLA2*, AtCslA3-CDS-1366F and AtCslA3-CDS-1433R for *AtCSLA3*, AtCslA9-CDS-1483F and AtCslA9-CDS-1550R for *AtCSLA9*, AtCslA10-CDS-1370F and AtCslA10-CDS-1429R for *AtCSLA10*, and At4G26410-Fwd and At4G26410-Rev for *At4G26410*. PCR was conducted with a 7500 Fast Real-Time PCR System (Applied Biosystems) and data were analyzed with the 7500 Fast System SDS 1.4 software (Applied Biosystems). The PCR threshold cycle number of target genes was normalized to that of the reference gene *At4G26410* (Czechowski *et al*., 2005) to calculate the relative transcript levels. Relative expression of target genes in *msr* mutants was presented relative to that in the WT.

**Gene cloning and construct generation**

The coding region with (CDS+) or without (CDS-) a stop codon was amplified by RT-PCR from RNA isolated from fenugreek endosperm at 30 days post anthesis (DPA) for *TfMSR*, by PCR from the cDNA clone U20773 [*Arabidopsis* Biological Resource Center (ABRC), http://abrc.osu.edu/] for *AtMSR1*, and by RT-PCR from RNA isolated from WT Col-0 seedlings for *AtMSR2*. The PCR primers used are: TfMSR+Start and TfMSR+Stop for *TfMSR* CDS+, TfMSR+Start and TfMSR-Stop for *TfMSR* CDS-, At3g21190+Start and At3g21190+Stop for *AtMSR1* CDS+, At3g21190+Start and At3g21190-Stop for *AtMSR1* CDS-, At1g51630+Start and At1g51630+Stop for *AtMSR2* CDS+, and At1g51630+Start and At1g51630-Stop for *AtMSR2* CDS-. The PCR products were cloned into the Gateway entry vector pENTR/D-TOPO using the pENTR Directional TOPO Cloning Kit (Invitrogen).

To generate an N-terminus or C-terminus GFP-tagged *MSR* construct (35S_pro_:*GFP-MSR* or 35S_pro_:*MSR-GFP*), the *MSR* coding region with or without a stop codon was recombined into the Gateway destination vector pK7WGF2 or pK7FWG2 (Karimi *et al*., 2002) using the Gateway LR Clonase II Enzyme Mix (Invitrogen). To generate a MSR overexpressing contruct, the *MSR* coding region with a stop codon was recombined into the Gateway destination vector pH2GW7 (Karimi *et al*., 2002).

To generate an *AtMSR* promoter-*GUS* transcriptional fusion construct (AtMSR_pro_:*GUS*), a promoter region was amplified by PCR from a BAC clone. Primers At3g21190-Prom-1480F and At3g21190-Prom-1R were used to amplify a 1.5-kb promoter region of *AtMSR1* from the BAC clone MIXL8 (ABRC), and At1g51630-Prom-1998F and At1g51630-Prom-56R to amplify a 1.9-kb promoter region of *AtMSR2* from the BAC clone F19C24 (ABRC). The promoter fragments were cloned into the vector pENTR/D-TOPO (Invitrogen), and subsequently recombined into the Gateway destination vector pKGWFS7 (Karimi *et al*., 2002) by LR reactions.

**GUS staining**

Each AtMSR_pro_:*GUS* construct was transformed into *Agrobacterium* GV3101 by electroporation. The GV3101 strain containing the construct was used to transform WT Col-0 plants by the floral dip approach (Clough and Bent, 1998). Positive transformants were selected by germinating the T1 seeds on the MS medium containing 500 µg/ml vancomycin and 50 µg/ml kanamycin. The T3 homozygous seedlings or plants with a single copy of the transgene were used for GUS staining according to Kim *et al*. (2006) with modifications.

Whole seedlings (4 and 10-day-old) or tissues (leaves, flower bundles, siliques and manually cut stem sections) from 7-week-old plants were harvested into cold 90% (v/v) acetone on ice. Samples were fixed at room temperature for 20 min, and washed in staining buffer (0.5% triton X-100, 2 mM K_3_Fe(CN)_6_, 100 mM Na_2_HPO_4_, pH7.2) three times on ice. The fixed samples were soaked in staining buffer plus 2 mM X-Gluc (5-Bromo-4-chloro-3-indoxyl-beta-D-glucuronide cyclohexylammonium salt), briefly vacuum infiltrated, and incubated at 37°C with gentle agitation overnight. The samples were then cleared according to Malamy and Benfey (1997). They were observed under a Zeiss Stemi 2000-C stereoscope (Carl Zeiss, http://www.zeiss.de/) or a Zeiss Axio Imager.M1 light microscope with a dark field filter, and pictures were taken using a Zeiss AxioCam MRc digital camera attached to the microscopes.

**Bioinformatic analysis**

A conserved domain of TfMSR was predicted by searching the Pfam database (Finn *et al*., 2010). Search of sequenced plant genome databases for GT65R family members was performed using BLASTP and TBLASTN programs with the deduced TfMSR amino acid sequence as a query, in combination with keyword search of gene ontologies or annotations using “PF10250” (Pfam identity number of the O-FucT family) as a search term. The genome data were obtained from the following sources: the *Arabidopsis* Information Resource (TAIR, http://www.arabidopsis.org/) for *Arabidopsis*, the Rice Genome Annotation Project (http://rice.plantbiology.msu.edu/) for rice, and the Joint Genome Institute (JGI, http://genome.jgi-psf.org/) for other sequenced species.

A phylogenetic tree was constructed using MEGA5.05 (Tamura *et al*., 2011) with the maximum likelihood method and 1000 bootstrap replications. Due to great variations in the amino-terminal region, only sequences at the conserved domain of TfMSR and 39 *Arabidopsis* GT65R proteins were used for phylogenetic analysis.

Cellular localization of proteins was predicted by TargetP (Emanuelsson *et al*., 2007). Prediction of transmembrane domains was performed using TMHMM2.0 (http://www.cbs.dtu.dk/services/TMHMM/) and ARAMEMNON (http://aramemnon.botanik.uni-koeln.de/). Search for distant protein structural homologues was conducted using the FUGUE program (Shi *et al*., 2001). The expression patterns of the putative *Arabidopsis* *O-FucT* genes were visualized by the *Arabidopsis* eFP Browser (Winter *et al*., 2007) based on the publically available AtGenExpress microarray data (Schmid *et al*., 2005).

**Clough, S.J. and Bent, A.F.** (1998) Floral dip: a simplified method for Agrobacterium-mediated transformation of *Arabidopsis thaliana*. *Plant J*. **16**, 735-743.

**Czechowski, T., Stitt, M., Altmann, T., Udvardi, M.K. and Scheible, W.R.** (2005) Genome-wide identification and testing of superior reference genes for transcript normalization in *Arabidopsis*. *Plant Physiol*. **139**, 5-17.

**Emanuelsson, O., Brunak, S., von Heijne, G. and Nielsen, H.** (2007) Locating proteins in the cell using TargetP, SignalP and related tools. *Nat. Protoc*. **2**, 953-971.

**Finn, R.D., Mistry, J., Tate, J. *et al.*** (2010) The Pfam protein families database. *Nucleic Acids Res*. **38**, D211-222.

**Karimi, M., Inze, D. and Depicker, A.** (2002) GATEWAY vectors for Agrobacterium-mediated plant transformation. *Trends Plant Sci*. **7**, 193-195.

**Kim, K.-W., Franceschi, V.R., Davin, L.B. and Lewis, N.G.** (2006) β-glucuronidase as reporter gene: advantages and limitations. *Methods Mol. Biol*. **323**, 263-273.

**Malamy, J.E. and Benfey, P.N.** (1997) Organization and cell differentiation in lateral roots of *Arabidopsis thaliana*. *Development*, **124**, 33-44.

**Schmid, M., Davison, T.S., Henz, S.R., Pape, U.J., Demar, M., Vingron, M., Scholkopf, B., Weigel, D. and Lohmann, J.U.** (2005) A gene expression map of *Arabidopsis thaliana* development. *Nat. Genet*. **37**, 501-506.

**Shi, J.Y., Blundell, T.L. and Mizuguchi, K.** (2001) FUGUE: Sequence-structure homology recognition using environment-specific substitution tables and structure-dependent gap penalties. *J. Mol. Biol.* **310**, 243-257.

**Tamura, K., Peterson, D., Peterson, N., Stecher, G., Nei, M. and Kumar, S.** (2011) MEGA5: molecular evolutionary genetics analysis using maximum likelihood, evolutionary distance, and maximum parsimony methods. *Mol. Biol. Evol*. **28**, 2731-2739.

**Wang, Y., Alonso, A.P., Wilkerson, C.G. and Keegstra, K.** (2012) Deep EST profiling of developing fenugreek endosperm to investigate galactomannan biosynthesis and its regulation. *Plant Mol. Biol*. **79**, 243-258.

**Winter, D., Vinegar, B., Nahal, H., Ammar, R., Wilson, G.V. and Provart, N.J.** (2007) An "Electronic Fluorescent Pictograph" browser for exploring and analyzing large-scale biological data sets. *PLoS One*, **2**, e718.
