# Supplementary material for: Identification of an additional protein involved in mannan biosynthesis
Source: Plant J. 2012 Oct 19;73(1):105–17. doi: 10.1111/tpj.12019 (PMC3558879; doi:10.1111/tpj.12019)
Supplement: Supplementary file 18 [file tpj0073-0105-SD8.doc]

**Supporting Information legends**

**Figure S1.** Sequence alignment of TfMSR, AtMSR1 and AtMSR2. Residues identical in at least two of the three sequences are highlighted in black background, and similar residues in grey background. A predicted transmembrane domain and the DUF246 domain are indicated by a red line and blue lines, respectively. A large C-terminal globular region spanning the DUF246 domain was predicted by TMHMM 2.0 (http://www.cbs.dtu.dk/services/TMHMM/) to be located in the Golgi lumen, and the probabilities for prediction of a transmembrane domain by TMHMM 2.0 are 0.5 for TfMSR, 0.9 for AtMSR1 and 0.8 for AtMSR2.

**Figure S2.** Expression of *AtMSR1* in comparison with *AtCSLA* genes summarized from previously published microarray data. (a) Expression of *AtMSR1* from publicly available AtGenExpress microarray data (Schmid *et al*., 2005) visualized by the *Arabidopsis* eFP Browser (Winter *et al*., 2007). (b) Expression of *AtMSR1* and *AtCSLA* genes in isolated guard cells and mesophyll cells (Yang *et al*., 2008). (c) Expression of *AtMSR1* and *AtCSLA* genes in trichomes and in the shoot without trichomes (Marks *et al*., 2009). The error bar represents standard deviation of biological replicates. (d) Expression *AtMSR1* and *AtCSLA9* in trichomes (Tri) and the leaf without trichomes (Lf w/o Tri) (Jakoby *et al*., 2008).

**Figure S3.** Quantitation of PACE band intensity. Ratio of band intensity of wild-type (WT) mannanase digested AIR compared to *MSR* mutant lines. Refer to Figure 7 for location of band “a” and “b”. The error bars represent the standard deviation of 3 biological replicates, with 2 technical replicates for each. The asterisk indicates statistically significant difference relative to the WT (Student’s t-test p value < 0.05). *1-1*, *msr1-1*; *2-2*, *msr2-2*; *1-1 2-2*, *msr1-1 msr2-2* double mutant.

**Figure S4.** Quantitative RT-PCR analysis of *MSR* mutants. (a) Expression of *AtMSR2* in *msr1* mutants and of *AtMSR1* in *msr2* mutants relative to that in the wild type (WT). (b) Expression of four *Arabidopsis* *CSLA* genes in the WT and *msr1-1 msr2-1* (*1-1 2-1*) and *msr1-1 msr2-2* (*1-1 2-2*) double mutants. The expression level was normalized to that of the reference gene *At4G26410* and presented relative to the level of *AtCSLA3* in the WT. The error bars represent the standard deviation of 3 biological replicates, with 2 technical replicates for each. The asterisk indicates statistically significant difference relative to the WT (Student’s t-test p value < 0.05).

**Figure S5**. Phylogenetic tree of TfMSR and the *Arabidopsis* GT65R proteins. Amino acid sequences in the GT65R conserved domain were used for tree construction. The tree is drawn to scale, and the scale bar shows variation represented by number of substitutions per site. The number at each node indicates a bootstrap value as percentage. The sequences of human POFUT1 (HsPOFUT1, accession number Q9H488 from the UniProtKB/Swiss-Prot database) and POFUT2 (HsPOFUT2, accession number Q9Y2G5) as well as *Drosophila* POFUT1 (DmPOFUT1, accession number Q9V6X7) and POFUT2 (DmPOFUT2, accession number Q9W589) are also included in the tree. The experimentally determined localizations for some of the *Arabidopsis* GT65R proteins (see Table S2 for references) are shown in parentheses. VM, vacuolar membrane. HsPOFUT1, DmPOFUT1 and At3g05320 belong to the GT65 family, and HsPOFUT2, DmPOFUT2, At1g17270, At1g53770 and At5g50420 belong to the GT68 family in the CAZy database (http://www.cazy.org/).

**Table S1.** Number of *GT65R* genes present in plant genomes

**Table S2.** Annotated *Arabidopsis* GT65R proteins

**Table S3.** Sequences of primers used for PCR.

**Methods S1.** Supplementary experimental procedures.

**References**

**Jakoby, M.J., Falkenhan, D., Mader, M.T., Brininstool, G., Wischnitzki, E., Platz, N., Hudson, A., Hulskamp, M., Larkin, J. and Schnittger, A.** (2008) Transcriptional profiling of mature *Arabidopsis* trichomes reveals that NOECK encodes the MIXTA-like transcriptional regulator MYB106. *Plant Physiol*. **148**, 1583-1602.

**Marks, M.D., Wenger, J.P., Gilding, E., Jilk, R. and Dixon, R.A.** (2009) Transcriptome analysis of *Arabidopsis* wild-type and gl3-sst sim trichomes identifies four additional genes required for trichome development. *Mol. Plant*, **2**, 803-822.

**Schmid, M., Davison, T.S., Henz, S.R., Pape, U.J., Demar, M., Vingron, M., Scholkopf, B., Weigel, D. and Lohmann, J.U.** (2005) A gene expression map of *Arabidopsis thaliana* development. *Nat. Genet*. **37**, 501-506.

**Winter, D., Vinegar, B., Nahal, H., Ammar, R., Wilson, G.V. and Provart, N.J.** (2007) An "Electronic Fluorescent Pictograph" browser for exploring and analyzing large-scale biological data sets. *PLoS One*, **2**, e718.

**Yang, Y., Costa, A., Leonhardt, N., Siegel, R.S. and Schroeder, J.I.** (2008) Isolation of a strong *Arabidopsis* guard cell promoter and its potential as a research tool. *Plant Methods*, **4**, 6.
